# Procalcitonin-Informed Decision-Making in Abdominal and Emergency Surgery: A WSES Position Paper

**DOI:** 10.3390/antibiotics15101042

**Published:** 2026-10-21

**Authors:** Fausto Catena, Belinda De Simone, Igor A. Kryvoruchko, Salomone Di Saverio, Marcelo A. F. Ribeiro, Timothy Craig Hardcastle, Arda Işık, Orestis Ioannidis, Mauro Podda, Marcello Di Martino, Chad G. Ball, Tal M. Hörer, Pablo Ottolino, Jung-Min Bae, Fernando J. Kim, Kemal Rasa, Vishal G. Shelat, Pradeep Navsaria, Agron Dogjani, Edward Tan, Francesca Dal Mas, Zsolt J. Balogh, Isidoro Di Carlo, Guixi Zhang, Jae Gil Lee, Dieter G. Weber, Marco Ceresoli, Gary Alan Bass, Fikri M. Abu-Zidan, Andrey Litvin, David Costa-Navarro, Edoardo Picetti, Ana María González Castillo, Andreas Hecker, Aleix Martínez-Pérez, Carlos A. Ordoñez, Angela Gurrado, Nikolaos Pararas, Carlos Augusto Gomes, Marja A. Boermeester, Gustavo P. Fraga, Emmanouil Pikoulis, Anastasia Pikouli, Jim Khan, Paula Ferrada, Boris E. Sakakushev, Michel P. J. Teuben, Tadeja Pintar Kaliterna, Krstina Doklestić Vasiljev, José Manuel Ramia, Andrew W Kirkpatrick, Kenji Inaba, Ernest E. Moore, Walter L. Biffl, Manu L. N. G. Malbrain, Gia Tomadze, Juan Carlos Puyana, Ari Leppäniemi, Michael Sugrue, Imtiaz Wani, Rifat Latifi, Haytham M. Kaafarani, Philip F. Stahel, Luca Ansaloni, Federico Coccolini, Massimo Sartelli

**Affiliations:** 1 World Society of Emergency Surgery, Institute for Research in Emergency General Surgery (IREGS), Via Cracovia 23, 40139 Bologna, Italy; faustocatena@gmail.com; 2 Department of Emergency and General Minimally Invasive Surgery, Level I Trauma Center, Bufalini Hospital, AUSL Romagna, Viale Ghirotti 286, 47521 Cesena, Italy; 3 Department of Theoretical and Applied Sciences, eCampus University, Novedrate, 22060 Como, Italy; 4 Department of Surgery No. 2, Kharkiv National Medical University, 61022 Kharkiv, Ukraine; ikryvoruchko60@gmail.com; 5 Department of General Surgery, Madonna del Soccorso Hospital, 63074 San Benedetto del Tronto, Italy; 6 Department of Surgery, University of Maryland School of Medicine, Baltimore, MD 21201, USA; mfribeiro@som.umaryland.edu; 7 R Adams Cowley Shock Trauma Center, Baltimore, MD 21201, USA; 8 Trauma and Burns Service, Department of Surgery, Nelson R. Mandela School of Medicine, University of KwaZulu-Natal, Durban 4013, South Africa; hardcastle@ukzn.ac.za; 9 Inkosi Albert Luthuli Central Hospital, Durban 4091, South Africa; 10 Department of General Surgery, School of Medicine, Istanbul Medeniyet University, 34700 Istanbul, Türkiye; kararda@yahoo.com; 11 Fourth Department of Surgery, School of Medicine, Aristotle University of Thessaloniki, 54124 Thessaloniki, Greece; 12 George Papanikolaou General Hospital, 54124 Thessaloniki, Greece; 13 Department of Surgical Sciences, University of Cagliari, 09042 Cagliari, Italy; mauro.podda@unica.it; 14 Department of Health Sciences, University of Piemonte Orientale, 28100 Novara, Italy; marcello.dimartino@uniupo.it; 15 Division of General Surgery, University Hospital Maggiore della Carità, 28100 Novara, Italy; 16 Department of Surgery, University of Calgary, Calgary, AB T2N 1N4, Canada; ball.chad@gmail.com; 17 Department of Hepatobiliary and Pancreatic Surgery, Trauma and Acute Care Surgery, Foothills Medical Centre, Calgary, AB T2N 2T9, Canada; 18 Department of Cardiothoracic and Vascular Surgery, Faculty of Medicine and Health, Örebro University, 70182 Örebro, Sweden; 19 Department of Surgery, Faculty of Medicine and Health, Örebro University, 70182 Örebro, Sweden; 20 Trauma, Emergency and Abdominal Wall Unit, Hospital Dr. Sótero del Río, Av Concha y Toro 3459, Puente Alto Santiago 8150215, Chile; 21 Department of Surgery, Yeungnam University Medical Center, Yeungnam University College of Medicine, Daegu 42415, Republic of Korea; 22 Department of Urology, AdventHealth Riverview, Riverview, FL 33878, USA; 23 Department of General Surgery, Anadolu Medical Center Hospital, 41400 Kocaeli, Türkiye; kemrasa@gmail.com; 24 Department of General Surgery, Tan Tock Seng Hospital, Singapore 308433, Singapore; vgshelat@gmail.com; 25 Trauma Centre, Groote Schuur Hospital, Cape Town 7700, South Africa; 26 Department of Surgery, University of Cape Town, Cape Town 7700, South Africa; 27 Department of Surgery, University Hospital of Trauma, 1000 Tirana, Albania; 28 Faculty of Medicine, University of Medicine, 1010 Tirana, Albania; 29 Department of Surgery, Radboud University Medical Center, 6625 GA Nijmegen, The Netherlands; edward.tan@radboudumc.nl; 30 Venice School of Management, Ca’ Foscari University of Venice, 30123 Venice, Italy; 31 Collegium Medicum, SAN University, 90519 Łódź, Poland; 32 Department of Traumatology, John Hunter Hospital, Newcastle, NSW 2308, Australia; 33 School of Medicine and Public Health, University of Newcastle, Newcastle, NSW 2308, Australia; 34 Department of Surgical Sciences and Advanced Technologies, University of Catania, 95131 Catania, Italy; 35 Cannizzaro Hospital, 95131 Catania, Italy; 36 Department of Surgery, School of Medicine, The Chinese University of Hong Kong, Shenzhen 518172, China; zhangguixi@cuhk.edu.cn; 37 Department of Surgery, Ewha Womans University College of Medicine, Ewha Womans University Mokdong Hospital, Seoul 07985, Republic of Korea; jakii71@gmail.com; 38 Department of General Surgery, Royal Perth Hospital, Perth, WA 6000, Australia; 39 University of Western Australia, Perth, WA 6009, Australia; 40 Curtin University, Perth, WA 6845, Australia; 41 University of Newcastle, Callaghan, NSW 2308, Australia; 42 School of Medicine and Surgery, University of Milano-Bicocca, 20900 Monza, Italy; 43 Center for Emergency Surgical Outcomes Research, Division of Traumatology, Surgical Critical Care and Emergency Surgery, Philadelphia, PA 19104, USA; 44 Department of Biostatistics, Epidemiology and Informatics, Perelman School of Medicine, University of Pennsylvania, Philadelphia, PA 19104, USA; 45 Trauma and Emergency Research Group, Department of Surgery, College of Medicine and Health Sciences, United Arab Emirates University, Al Ain P.O. Box 15551, United Arab Emirates; fabuzidan@uaeu.ac.ae; 46 Department of Surgical Diseases No. 3, Gomel State Medical University, 246000 Gomel, Belarus; aalitvin@gmail.com; 47 University Clinic, 246000 Gomel, Belarus; 48 Department of General and Digestive Surgery, Elda General University Hospital, 03600 Elda, Spain; 49 Department of Anesthesia and Intensive Care, Parma University Hospital, 43121 Parma, Italy; edoardopicetti@hotmail.com; 50 Trauma and Emergency Surgery Unit, Department of General Surgery, Hospital del Mar, 08003 Barcelona, Spain; 51 Department of General and Thoracic Surgery, University Hospital of Giessen, 35392 Giessen, Germany; 52 Department of Surgery, University of Valencia, 46010 Valencia, Spain; 53 Division of Trauma and Acute Care Surgery, Department of Surgery, Fundación Valle del Lili, Cali 760032, Colombia; 54 Universidad del Valle, Cali 760032, Colombia; 55 Universidad Icesi, Cali 760032, Colombia; 56 University Hospital of Bari, 70124 Bari, Italy; angelagurrado.uniba@gmail.com; 57 Third Department of Surgery, National and Kapodistrian University of Athens, Attikon University Hospital, 10679 Athens, Greece; npararas@gmail.com (N.P.);; 58 Department of Surgery, Hospital Universitário Therezinha de Jesus, Faculdade de Ciências Médicas e da Saúde de Juiz de Fora—SUPREMA, Juiz de Fora 36033-003, Brazil; caxiaogomes@gmail.com; 59 Department of Surgery, Amsterdam UMC, University of Amsterdam, 1105 AZ Amsterdam, The Netherlands; 60 Amsterdam Gastroenterology Endocrinology and Metabolism, 1105 AZ Amsterdam, The Netherlands; 61 Division of Trauma Surgery, Department of Surgery, School of Medical Sciences, Universidade Estadual de Campinas, Campinas 70124, Brazil; 62 University of Portsmouth, Portsmouth PO1 2UP, UK; 63 Portsmouth Hospitals University NHS Trust, Portsmouth PO6 3FT, UK; 64 Department of Surgery, Inova Health, Fairfax, VA 22033, USA; 65 Department of Propedeutics of Surgical Diseases, Medical University of Plovdiv, 4002 Plovdiv, Bulgaria; bsakakushev@gmail.com; 66 First Clinic of General Surgery, University Hospital St George, 4001 Plovdiv, Bulgaria; 67 Department of Traumatology, University Hospital Zurich, 8091 Zürich, Switzerland; 68 Harald Tscherne Laboratory for Orthopaedic and Trauma Research, University Hospital Zurich, 8091 Zürich, Switzerland; 69 Department of Abdominal Surgery, Division of Surgery, University Medical Centre Ljubljana, 1000 Ljubljana, Slovenia; 70 Faculty of Medicine, University of Belgrade, 11000 Belgrade, Serbia; krstinadoklestic@gmail.com; 71 Emergency Surgical Department, University Clinical Center of Serbia, 11000 Belgrade, Serbia; 72 Department of Surgery, Dr. Balmis General University Hospital, 03010 Alicante, Spain; jose_ramia@hotmail.com; 73 Miguel Hernández University, 03202 Alicante, Spain; 74 Departments of Surgery and Critical Care Medicine, Cumming School of Medicine, University of Calgary, Calgary, AB T2N 1N4, Canada; 75 Foothills Medical Centre, Calgary, AB T2N 2T9, Canada; 76 Department of Surgery, Keck School of Medicine, University of Southern California, Los Angeles, CA 90089, USA; 77 Los Angeles General Medical Center, Los Angeles, CA 90033, USA; 78 Department of Surgery, Denver Health Medical Center, Denver, CO 80204, USA; 79 University of Colorado School of Medicine, Denver, CO 80045, USA; 80 Department of Surgery, Scripps Clinic and Scripps Clinic Medical Group, San Diego, CA 92037, USA; 81 First Department of Anaesthesiology and Intensive Therapy, Medical University of Lublin, 20059 Lublin, Poland; 82 International Fluid Academy, 3360 Bierbeek, Belgium; 83 Department of Surgery, Tbilisi State Medical University, 0186 Tbilisi, Georgia; g.tomadze@tsmu.edu; 84 Pineo Medical Ecosystem, 0186 Tbilisi, Georgia; 85 Institute of Global Surgery, School of Population Health, Royal College of Surgeons in Ireland, D02 X0N1 Dublin, Ireland; 86 Department of Abdominal Surgery, Helsinki University Hospital, 00290 Helsinki, Finland; 87 University of Helsinki, 00100 Helsinki, Finland; 88 Department of Surgery, RUMC Medical School, 10450 Georgetown, Malaysia; michaelesugrue@rumc.edu.my; 89 Department of Surgery, Government Gousia Hospital, Directorate of Health Services, Srinagar 190017, India; 90 Department of Surgery, Westchester Medical Center, Valhalla, NY 10595, USA; 91 New York Medical College, Valhalla, NY 10595, USA; 92 Division of Trauma, Emergency Surgery and Surgical Critical Care, Department of Surgery, Massachusetts General Hospital, Boston, MA 02114, USA; 93 Harvard Medical School, Boston, MA 02115, USA; 94 Department of Surgery, Brody School of Medicine, East Carolina University, Greenville, NC 27834, USA; 95 College of Osteopathic Medicine, Rocky Vista University, Parker, CO 80134, USA; 96 Mission Health, HCA Healthcare North Carolina Division, Asheville, NC 28803, USA; 97 Department of General Surgery I, University of Pavia, 27100 Pavia, Italy; 98 Fondazione IRCCS Policlinico San Matteo, 27100 Pavia, Italy; 99 General, Emergency and Trauma Surgery Department, Pisa University Hospital, 56100 Pisa, Italy; 100 Department of Surgery, Macerata Hospital, 62100 Macerata, Italy; massimosartelli@gmail.com

**Keywords:** procalcitonin, abdominal sepsis, intra-abdominal infection, source control, antimicrobial stewardship, antibiotic de-escalation, antibiotic discontinuation, secondary peritonitis, relaparotomy, anastomotic leak, acute pancreatitis, trauma surgery

## Abstract

**Background:** Procalcitonin (PCT) may support decision-making in abdominal and emergency surgery when infection is difficult to distinguish from non-infectious inflammation. Its interpretation remains challenging because clinical settings, sampling schedules, and proposed thresholds vary. This WSES position paper examines the evidence across six clinical scenarios to provide a practical framework for PCT-informed decision-making. **Methods:** A structured literature review covering January 1980 to December 2025, with targeted updates through August 2026, informed six PICO-based clinical questions. Evidence appraisal drew on selected GRADE domains, including consistency, directness, and precision. Recommendations were developed through iterative expert review and consensus. **Results:** The panel formulated six conditional recommendations addressing intra-abdominal infection diagnosis, antibiotic discontinuation after source control, suspected source-control failure, infected pancreatic necrosis, infectious complications after trauma, and postoperative anastomotic leak. The panel’s qualitative assessment rated evidence certainty as moderate for antibiotic discontinuation and the pancreatitis domain, and low for the remaining domains. Serial PCT trends may complement clinical assessment in selected patients. Evidence for reduced antibiotic exposure with predefined PCT algorithms is more established than evidence that PCT-informed imaging, reintervention, or discharge decisions improve patient outcomes. **Conclusions:** PCT-informed decision-making in abdominal surgery may contribute to a more individualized approach to emergency surgical care and precision emergency surgery. When interpreted as part of serial assessment and integrated with clinical judgement, organ-dysfunction trends, microbiology, imaging, and evaluation of source-control adequacy, PCT may support individualized antibiotic discontinuation, potentially earlier recognition of an unfavourable clinical course, and more timely diagnostic and source-control reassessment. Current evidence does not support universal thresholds or routine serial testing in all surgical patients. Biomarker kinetics should strengthen, but not replace, surgical reasoning or independently determine antimicrobial, imaging, or interventional decisions. Future research should validate setting-specific thresholds and sampling schedules, establish whether PCT provides actionable information before clinical deterioration, and evaluate the clinical effects and cost-effectiveness of PCT-informed pathways.

## 1. Background

Procalcitonin (PCT) is a 116-amino-acid precursor of calcitonin that is normally produced by thyroid parafollicular C cells and circulates at very low concentrations in healthy individuals, generally below 0.1 ng/mL, although reference ranges vary according to the assay used [1,2,3]. During bacterial infection, PCT expression is upregulated in multiple extra-thyroidal tissues in response to microbial endotoxins and pro-inflammatory cytokines, including interleukin-1, interleukin-6, and tumour necrosis factor-α [1].

The molecular sequence of human preprocalcitonin was characterized by Le Moullec and colleagues in the 1980s, while the association between elevated PCT concentrations and severe bacterial infection or sepsis was subsequently reported by Assicot and colleagues in the early 1990s [2,4]. PCT responses may be attenuated during viral infections, partly through interferon-γ-mediated suppression of PCT production. Its kinetics are clinically relevant: PCT usually increases within 4–6 h after bacterial stimulation, peaks at approximately 24 h, and declines as bacterial infection is controlled [4,5,6]. Serial changes may therefore provide more clinically relevant information than isolated measurements.

Intra-abdominal infections (IAIs) and abdominal sepsis remain major causes of morbidity and mortality in surgical patients and impose a substantial global burden. A recent analysis of the MICROBE database estimated that IAI-related deaths increased from 394,500 (95% uncertainty interval [UI], 361,200–427,800) in 1990 to 646,359 (95% UI, 585,349–707,369) in 2021, when IAIs accounted for 5.51% of infection-related deaths worldwide. Approximately 329,000 deaths in 2021 were associated with antimicrobial resistance, emphasizing the close relationship between the burden of IAIs, appropriate antimicrobial treatment, effective source control, and antimicrobial stewardship [7]. In the prospective nationwide Italian IRIS study, which included 784 patients with complicated IAIs requiring intensive care admission, in-hospital mortality was 23.9%; septic shock and sepsis accounted for 36.2% and 35.9% of ICU admissions, respectively. Adequate source control was achieved in 61.5% of patients, reoperation was required in 21%, and empirical antimicrobial therapy was administered to more than 80%, with median treatment durations ranging from 8.1 to 19.3 days [8].

Secondary peritonitis, infected pancreatic necrosis, postoperative intra-abdominal abscess, anastomotic leak, and infectious complications after trauma may rapidly progress to septic shock and multiple organ dysfunction when diagnosis, antimicrobial treatment, or source control is delayed. Their recognition is particularly challenging in postoperative, critically ill, and trauma patients, in whom bacterial infection and sterile inflammation may coexist and clinical findings and conventional inflammatory markers may be difficult to interpret.

PCT has consequently been investigated as an adjunctive biomarker across the surgical pathway, including the diagnosis and reassessment of intra-abdominal infections, evaluation of the response to source control, recognition of postoperative infectious complications, and antimicrobial stewardship [9]. PCT-guided antibiotic discontinuation strategies, particularly those evaluated in general sepsis and intensive care populations, have commonly used an absolute concentration below approximately 0.5 ng/mL or a decrease of at least 80% from the peak value in clinically improving patients [10].

Available evidence is difficult to translate into consistent decisions across abdominal surgical settings because studies differ in patient populations, clinical questions, measurement timing, and proposed thresholds. Surgeons therefore need clearer, scenario-specific guidance on when to measure PCT, how to interpret its trajectory, and which clinical decisions it may inform.

This WSES position paper addresses that need by critically appraising and integrating the evidence across six clinical scenarios: diagnosis of intra-abdominal infection, antibiotic discontinuation after adequate source control, suspected source-control failure, infected pancreatic necrosis, infectious complications after trauma, and postoperative anastomotic leak. For each scenario, we formulate a practical recommendation specifying when PCT measurements or serial trends may be useful, how they should be interpreted alongside clinical assessment, other laboratory markers, microbiology, and imaging, and which decisions they may support. Our aim is to provide surgical teams with an evidence-informed framework for incorporating PCT into practice while identifying thresholds and management strategies that still require prospective validation.

## 2. Methods

A WSES expert panel identified six clinically relevant domains: diagnosis and reassessment of intra-abdominal infection; antibiotic discontinuation after adequate source control; suspected source-control failure and need for repeat intervention; acute pancreatitis and infected pancreatic necrosis; infectious complications after trauma; and early detection or exclusion of postoperative anastomotic leak. These domains were selected for their relevance to surgical practice, antimicrobial stewardship, and source-control decision-making.

The paper addresses adult patients with abdominal surgical conditions, patients undergoing emergency or elective abdominal surgery, and adult trauma patients at risk of infectious complications. “PCT-informed decision-making” describes the integration of PCT measurements and serial trajectories with clinical findings, organ-dysfunction trends, conventional laboratory markers, microbiology, imaging, and assessment of source-control adequacy. “PCT-guided” is reserved for studies evaluating a prespecified biomarker threshold or kinetic rule linked to a recommended management action. Neither term implies that PCT overrides clinical judgement.

Two designated researchers formulated the clinical questions according to the Population, Intervention or Index test, Comparator, and Outcome (PICO) framework [11] and conducted the corresponding literature analysis. The intervention or index test was PCT measurement, either as an isolated value or as a serial trajectory. Comparators included clinical assessment, conventional inflammatory biomarkers, organ-dysfunction scores, microbiology, imaging, and usual care without incorporation of PCT. Outcomes included diagnostic and prognostic performance, antimicrobial exposure and discontinuation, recognition of persistent infection or inadequate source control, repeat intervention, postoperative infectious complications, morbidity, mortality, and length of stay. The complete PICO questions are presented in Table 1.

A structured literature search following PRISMA 2020 principles [12] was conducted in PubMed/MEDLINE, Embase, Scopus, and the Cochrane Library for articles published from January 1980 to December 2025. Search terms combined “procalcitonin” with terms related to the six domains, including “intra-abdominal infection”, “abdominal sepsis”, “secondary peritonitis”, “tertiary peritonitis”, “source control”, “reintervention”, “relaparotomy”, “antibiotic discontinuation”, “antibiotic de-escalation”, “antimicrobial stewardship”, “acute pancreatitis”, “infected pancreatic necrosis”, “trauma”, “anastomotic leak”, “postoperative infection”, and “gastrointestinal surgery”. Reference lists of relevant publications were also examined. Targeted evidence updates through August 2026 were considered when directly relevant. This focused review was intended to inform the position statements rather than provide an exhaustive systematic synthesis.

The review considered randomized trials, observational and diagnostic-accuracy studies, systematic reviews, meta-analyses, guidelines, and relevant consensus documents. Evidence from the target surgical or trauma populations was distinguished from indirect evidence obtained in general sepsis, mixed intensive care, or other populations. Exclusively paediatric or preclinical studies and publications without relevance to the predefined questions were not considered. Associations, diagnostic or prognostic performance, and effects of PCT-based management strategies were distinguished when interpreting study findings. The key selected studies supporting each clinical domain are summarized in Supplementary Tables S1–S6.

The panel used a qualitative, GRADE-informed approach to appraise the supporting evidence [13]. This appraisal drew on study design, consistency of findings, directness to the target population and clinical decision, and precision of reported estimates. The certainty labels reported represent the panel’s qualitative judgments based on these considerations.

Evidence summaries and draft statements were reviewed by the Steering Committee and subsequently circulated to the WSES expert panel. Comments submitted by email and through tracked manuscript revisions were collated by the coordinating authors and incorporated through iterative revision. No formal Delphi process, anonymous voting, or prespecified numerical agreement threshold was used. Final agreement was reached through resolution of substantive comments and panel approval. Table 2 summarizes the six recommendations, the panel’s judgments of evidence certainty, and the strength and direction of the recommendations.

## 3. Results

The six recommendations are organized by clinical scenario and reflect three overlapping functions of PCT: clinical assessment, antimicrobial stewardship, and source-control reassessment. Recommendations 1, 4, 5, and 6 primarily address the assessment of suspected infection or complications; Recommendation 2 addresses antibiotic discontinuation after adequate source control; and Recommendation 3 addresses reassessment for possible source-control failure. In some scenarios, particularly infected pancreatic necrosis and anastomotic leak, PCT findings may inform more than one of these functions. Each section specifies the clinical decision that PCT may inform within that scenario.

### 3.1. PCT for Diagnosis of Intra-Abdominal Infections

#### 3.1.1. Key Clinical Question 1

In adult patients with suspected intra-abdominal infection or abdominal sepsis, does procalcitonin measurement, alone or as part of serial biomarker assessment, improve diagnostic accuracy or clinical decision-making compared with clinical assessment, conventional laboratory markers, microbiology, and imaging without PCT?

#### 3.1.2. Recommendation 1

In adult patients with suspected intra-abdominal infection or abdominal sepsis and persistent diagnostic uncertainty, particularly those at risk of complicated or systemic infection, the WSES panel suggests using procalcitonin as an adjunct to integrated clinical, laboratory, microbiological, and imaging assessment. Serial measurements may support reassessment in selected postoperative or critically ill patients. Procalcitonin should not be used as a stand-alone diagnostic test, and its use should not delay imaging, empirical antimicrobial treatment in unstable patients, or urgent source control. [Certainty of evidence: Low. Strength of recommendation: Conditional, in favour.]

#### 3.1.3. Summary of Evidence

IAIs are major causes of morbidity and mortality in abdominal surgery. Prompt diagnosis remains challenging because clinical manifestations may be subtle or non-specific, particularly in postoperative, critically ill, older, or immunocompromised patients. Conventional inflammatory markers, including white blood cell count and C-reactive protein (CRP), are widely available but have limited specificity, as they may be influenced by surgical trauma, tissue ischaemia, sterile inflammation, organ dysfunction, and other non-infectious conditions.

PCT is released in response to bacterial infection and has been investigated as a biomarker to support diagnostic assessment in severe bacterial infections, IAIs, and abdominal sepsis [10]. Its kinetic profile is clinically relevant: PCT usually rises within 4–6 h after bacterial stimulation, peaks within approximately 24 h, and decreases when infection is controlled and source control is effective [14]. Conversely, persistently elevated concentrations, failure to decline, or a secondary rise after an initial decrease may raise concern for ongoing infection, inadequate source control, or postoperative infectious complications. However, these patterns are not specific to an abdominal source and should always be interpreted within the clinical context.

Available evidence suggests that PCT may help distinguish bacterial infection from non-infectious systemic inflammation when interpreted together with clinical findings, physiological and organ-dysfunction parameters, conventional laboratory tests, microbiology, and imaging [10,14,15,16,17]. Nevertheless, evidence across heterogeneous IAI populations remains limited. In a recent study of 245 patients with acute abdominal conditions, PCT concentrations were higher in complicated disease, but the biomarker provided limited additional value over conventional inflammatory markers for initial diagnosis and severity grading [18]. Similarly, in a retrospective study including 3429 emergency department patients with IAIs, early PCT measurement was not independently associated with reductions in mortality or major complications [19]. These findings do not support its routine measurement in all patients with suspected or uncomplicated IAI.

Disease-specific evidence suggests that PCT may be more useful for identifying complicated or systemic disease than for diagnosing the underlying abdominal condition. In suspected acute appendicitis, a systematic review and meta-analysis reported low sensitivity for the overall diagnosis but greater specificity for complicated appendicitis [20]. A subsequent systematic review similarly found limited diagnostic value in uncomplicated appendicitis but a potential role in identifying gangrenous, necrotic, or perforated disease [21].

In acute diverticulitis, PCT interpreted together with computed tomography may help differentiate complicated from uncomplicated disease [22]. Elevated admission PCT has also been associated with complicated diverticulitis, need for surgery, and mortality, supporting a potential role in risk stratification rather than primary diagnosis [23].

In acute cholecystitis, a systematic review found that PCT may contribute to severity assessment and the prediction of difficult surgery or postoperative complications; however, the available evidence was limited by small samples, heterogeneous thresholds, and inconsistent outcome definitions [24].

In patients with secondary peritonitis or abdominal sepsis, PCT may provide complementary information regarding the presence and systemic severity of bacterial infection. Preoperative PCT and CRP have shown some ability to distinguish sepsis from a sterile systemic inflammatory response, although their postoperative diagnostic performance appears more limited [25]. A further meta-analysis suggested a possible diagnostic role for PCT in bacterial peritonitis; however, evidence derived from spontaneous bacterial peritonitis or non-surgical populations cannot be directly extrapolated to all surgical IAIs [26].

The interpretation of PCT is particularly complex after major abdominal surgery. Postoperative elevations may reflect the magnitude and type of surgical procedure and are not necessarily attributable to infection. In patients with infections following major abdominal surgery, CRP, but not PCT, was associated with antimicrobial response and clinical outcomes, while PCT concentrations were influenced by the type of operation, particularly liver surgery [27]. PCT should therefore be regarded as an adjunct to, rather than a substitute for, clinical evaluation, imaging, and microbiological assessment.

PCT thresholds vary across studies, and their interpretation depends on the timing of measurement, disease severity, renal dysfunction, previous surgery or trauma, and adequacy of source control. A low or decreasing PCT concentration should not be used to exclude an IAI, override clinical deterioration, or delay empirical antimicrobial treatment, imaging, or urgent source control. Conversely, elevated or increasing values cannot localise the infection or independently determine the need for intervention.

Overall, PCT may support diagnostic reassessment in selected patients with persistent diagnostic uncertainty, suspected complicated or systemic infection, discordant clinical, laboratory, and imaging findings, or a postoperative or critical illness-related inflammatory response that limits the interpretation of conventional markers. Serial trajectories may be more informative than isolated measurements, but they should be obtained when they address a defined clinical question. Routine PCT measurement in low-risk patients with an otherwise clear diagnosis is not supported by the available evidence.

### 3.2. PCT-Informed Antibiotic Discontinuation After Source Control

#### 3.2.1. Key Clinical Question 2

In adult patients receiving antimicrobial treatment for intra-abdominal infection after adequate source control, does PCT-guided antibiotic discontinuation reduce antibiotic exposure compared with fixed-duration or clinically guided therapy, without increasing treatment failure or infectious recurrence?

#### 3.2.2. Recommendation 2

In adult patients treated for intra-abdominal infection after adequate source control, the WSES panel suggests using serial procalcitonin trends together with clinical assessment to support antibiotic discontinuation within an antimicrobial stewardship pathway. A substantial decline from peak PCT concentrations, commonly ≥80%, or a low absolute value may support discontinuation in clinically improving patients, according to locally validated protocols and assay availability. [Certainty of evidence: Moderate. Strength of recommendation: Conditional, in favour.]

#### 3.2.3. Summary of Evidence

After adequate source control of a complicated intra-abdominal infection (cIAI), prolonged antimicrobial therapy is generally not recommended and may contribute to antimicrobial resistance, drug-related toxicity, Clostridioides difficile infection, fungal superinfection, and increased healthcare costs [28]. Although traditional antibiotic courses frequently ranged from 7 to 14 days, evidence supports substantially shorter treatment. In the STOP-IT randomized trial, approximately four days of fixed-duration antimicrobial therapy after adequate source control resulted in outcomes similar to those obtained with a longer course continued until two days after resolution of fever, leukocytosis, and ileus [29]. Current recommendations therefore support a short fixed-duration course, generally approximately four days, in clinically improving patients after adequate source control [28].

Within this framework, serial PCT measurements may support earlier antibiotic discontinuation after adequate source control, particularly when integrated with clinical response and antimicrobial stewardship protocols. Because PCT generally declines as systemic bacterial inflammation resolves, serial trajectories may help distinguish a favourable response from persistent or recurrent infection. Nevertheless, PCT should complement rather than replace clinical reassessment and evaluation of source-control adequacy [9,28].

Randomized trials and meta-analyses conducted predominantly in general sepsis and critically ill populations indicate that PCT-guided discontinuation strategies can reduce antibiotic exposure without a consistent increase in mortality or treatment failure [30,31,32,33]. More recent evidence confirms a reduction in antibiotic duration but has also suggested a possible increase in infection recurrence, reinforcing the need for cautious interpretation [34]. These findings are only indirectly applicable to surgical IAIs because the included populations, infection sources, PCT algorithms, stopping thresholds, and usual treatment durations are heterogeneous.

Direct evidence in patients undergoing surgery for cIAIs is more limited. Huang et al. evaluated a PCT-based algorithm in which antibiotics were discontinued after resolution of clinical signs when PCT was below 1.0 ng/mL or had decreased by at least 80% from the early postoperative value. The algorithm was associated with a shorter median duration of antimicrobial therapy than that observed in propensity-matched historical controls, without an apparent increase in adverse events [35]. However, the small sample, non-randomized design, and use of historical controls limit the certainty of this finding.

Maseda et al. similarly reported an approximately 50% reduction in antibiotic duration among surgical ICU patients with controlled cIAIs managed using a PCT-guided strategy, without evidence of worse clinical outcomes [35]. Nevertheless, this was a relatively small observational study and cannot establish the safety or superiority of the strategy across different surgical populations.

In a randomized trial of patients receiving postoperative antibiotics after surgery for peritonitis, PCT guidance did not significantly reduce antibiotic duration in the overall study population. A possible reduction was observed in the subgroup with peritonitis caused by gastrointestinal perforation, without an apparent increase in adverse outcomes; however, the study was underpowered to support firm subgroup conclusions [36].

The stopping criteria used in surgical studies have not been uniform. An 80% reduction from peak or early postoperative PCT values has been adopted in several algorithms, whereas absolute stopping thresholds have varied across studies and clinical settings [35,36,37]. The frequently cited threshold of 0.5 ng/mL derives largely from general sepsis and ICU algorithms and should not be considered universally validated for postoperative IAIs [30,32]. Renal dysfunction, major surgery, trauma, immunosuppression, and other causes of systemic inflammation may also alter PCT concentrations or kinetics.

Routine serial PCT measurement is unlikely to provide substantial additional benefit in clinically improving patients with adequate source control who are already receiving a recommended short fixed-duration course [28,29]. Its potential value may be greater in postoperative or critically ill patients in whom conventional inflammatory abnormalities persist despite clinical improvement, or when antimicrobial therapy would otherwise be prolonged despite adequate source control and no clinical evidence of ongoing infection.

PCT should not be used as the only criterion for antibiotic discontinuation. Low or decreasing values should not override persistent haemodynamic instability, organ dysfunction, clinical deterioration, or concern for inadequate source control. Conversely, persistent PCT elevation alone should not automatically result in prolonged antimicrobial therapy; it should prompt clinical reassessment and, when appropriate, investigation for an uncontrolled source [9,28]. Selection or de-escalation of antimicrobial agents should remain primarily guided by microbiological results, susceptibility patterns, infection site, clinical response, and local stewardship protocols.

Overall, direct surgical evidence suggests that PCT-guided algorithms may reduce antibiotic exposure in patients with cIAIs, although the available studies remain limited and heterogeneous [35,36,37,38]. Serial PCT trends may therefore support earlier antibiotic discontinuation in clinically improving patients after adequate source control, whereas routine biomarker monitoring is not required to implement an evidence-based short-course strategy [28,29].

### 3.3. PCT Trends for Suspected Failed Source Control and Repeat Source-Control Decision-Making

#### 3.3.1. Key Clinical Question 3

In adult patients after surgery for secondary peritonitis or abdominal sepsis, do serial PCT trends help identify failed source control or the need for reintervention compared with clinical assessment, conventional inflammatory markers, imaging, or standard postoperative monitoring alone?

#### 3.3.2. Recommendation 3

In adult patients with suspected tertiary peritonitis or persistent or recurrent abdominal sepsis following initial source control, the WSES panel suggests incorporating serial procalcitonin measurements into the clinical reassessment pathway to support early identification of failed source control and the need for repeat intervention.

Persistently elevated PCT values, failure to decline within 48–72 h after the index procedure, or a secondary rise should prompt targeted reassessment for ongoing intra-abdominal infection or other causes of an unfavourable postoperative course. Reassessment should include repeat imaging and consideration of additional source-control procedures, including percutaneous or endoscopic drainage, surgical reintervention, or relaparotomy, according to the patient’s clinical condition and the suspected source of infection. [Certainty of evidence: Low. Strength of recommendation: Conditional, in favour.]

#### 3.3.3. Summary of Evidence

Tertiary peritonitis is a persistent or recurrent intra-abdominal infection following treatment of secondary peritonitis despite antimicrobial therapy and attempted source control. It typically occurs in critically ill postoperative patients with ongoing sepsis or organ dysfunction and may result from persistent contamination, an undrained collection, anastomotic leak, bowel ischaemia, or another inadequately controlled source.

Early recognition of failed source control is essential. Delayed diagnosis may lead to septic shock, progressive organ dysfunction, and death, whereas unnecessary reintervention may expose patients to additional procedural or operative trauma, complications, prolonged intensive care stay, and increased healthcare costs [39]. The choice among continued observation, repeat imaging, percutaneous or endoscopic drainage, and surgical reintervention remains one of the most difficult decisions in emergency general surgery.

Postoperative recognition of failed source control can be challenging because clinical signs and conventional inflammatory markers may remain abnormal after major abdominal surgery, while early imaging findings may be inconclusive. PCT has therefore been investigated as a dynamic biomarker of persistent abdominal sepsis and response to source control [1,15,40,41,42,43]. Following effective treatment, PCT concentrations are expected to decline. Persistent elevation, failure to decline, or a secondary rise after an initial decrease may indicate an unfavourable course, although these patterns are not specific to failed abdominal source control.

Evidence specifically addressing PCT in tertiary peritonitis is limited. Available findings derive mainly from patients undergoing surgery for secondary peritonitis or abdominal sepsis and therefore provide indirect evidence for this setting. These studies evaluated whether early postoperative changes in PCT were associated with successful elimination of the septic focus. The PCT ratio, also termed the “focus index” (FI), is defined as:

FI = serum PCT on postoperative day 1/serum PCT on postoperative day 2

Both concentrations are measured in the same units following the index source-control operation. The ratio is dimensionless: values > 1 indicate decreasing PCT, values = 1 indicate no change, and values < 1 indicate increasing PCT. In a cohort of 104 patients, Novotny et al. [43] identified a cutoff of 1.03, with lower ratios indicating unsuccessful surgical treatment; sensitivity and specificity for identifying treatment failure were 95% and 63%, respectively. In a subsequent prospective validation study involving 234 patients with secondary peritonitis, Assfalg et al. [41] identified a cutoff of 1.1, with higher values associated with effective source control. Following the data-cleaning procedure reported by the authors, sensitivity and specificity for identifying successful focus elimination were 93% and 71%, respectively. In that study, the optimal timing for the first sample was 12–24 h after the index operation. These study-specific thresholds may inform postoperative reassessment but should be interpreted alongside clinical findings and should not serve as standalone criteria for reintervention.

In contrast, in a cohort of 101 patients with perioperative intra-abdominal infection and septic shock, Jung et al. found that neither a decrease in PCT to 0.5 ng/mL nor a reduction of at least 80% from the peak predicted treatment response with sufficient accuracy. Treatment failure included death related to the initial infection, repeat surgical or radiological source control, and new intra-abdominal infection [44]. Taken together, these studies suggest that PCT kinetics may contribute to the assessment of postoperative trajectory but cannot independently establish failed source control or the need for reintervention.

Absolute PCT values above 5 or 10 ng/mL and relative decreases of approximately 50% or 80% have also been investigated in general sepsis and heterogeneous critically ill populations as indicators of infection severity, treatment response, organ dysfunction, or prognosis [30,31,32,33,34,45,46,47]. These thresholds are not specific for an uncontrolled intra-abdominal source and have shown inconsistent predictive performance. In patients with suspected tertiary peritonitis, persistent elevation, failure to show a clear downward trajectory within 48–72 h after initial source control, or a secondary rise should therefore be interpreted as warning patterns requiring reassessment. Prospective studies specifically enrolling patients with tertiary peritonitis are needed to determine whether absolute or kinetic PCT thresholds can reliably identify failed source control and guide repeat intervention.

PCT has faster kinetics than CRP and may provide earlier information on the response to antimicrobial therapy and source control. When the postoperative course is difficult to interpret, failure of PCT to decline or a secondary rise may reveal an unfavourable trajectory before conventional inflammatory markers have clearly differentiated persistent infection from the expected postoperative response. Serial PCT measurements may therefore add clinically useful information to CRP, white blood cell count, organ-dysfunction trends, and imaging, particularly when early recognition of failed source control is critical [15,46,47]. Although PCT cannot localise the source of infection, its dynamic response makes it a useful early-warning biomarker for prompting timely reassessment.

In patients with suspected tertiary peritonitis or persistent or recurrent abdominal sepsis, an unfavourable PCT trajectory should prompt targeted reassessment for ongoing contamination, an undrained collection, anastomotic leak, bowel ischaemia, or another uncontrolled intra-abdominal source. Alternative infectious and non-infectious causes should also be considered. Reassessment should include repeat imaging when appropriate. If failed source control is confirmed or remains strongly suspected, additional source control may involve percutaneous or endoscopic drainage, minimally invasive surgical reintervention, or relaparotomy, according to the suspected source and the patient’s clinical condition [48].

Conversely, declining PCT values may provide supportive evidence of improvement in clinically stable patients but should not exclude persistent infection or justify deferring investigation when clinical deterioration, organ dysfunction, or radiological suspicion persists. Current recommendations and reviews similarly consider PCT a potentially useful adjunct for recognizing an unfavourable evolution and prompting further investigation or intervention, while acknowledging the limited directness and low certainty of the supporting evidence [28,48,49].

Overall, serial PCT trends provide clinically useful early information on the response to treatment and may facilitate timely recognition of an unfavourable course in patients with suspected tertiary peritonitis or persistent or recurrent abdominal sepsis. Whether PCT identifies failure earlier than standard assessment or improves outcomes when used to trigger imaging or reintervention remains uncertain. Persistent elevation, failure to decline within 48–72 h after initial source control, or a secondary rise should prompt targeted reassessment for an uncontrolled source and consideration of repeat source-control intervention.

### 3.4. PCT in Acute Pancreatitis and Infected Pancreatic Necrosis

#### 3.4.1. Key Clinical Question 4

In adult patients with acute pancreatitis, do PCT measurements improve identification of infected pancreatic necrosis and support antibiotic or interventional management compared with clinical assessment, conventional biomarkers, imaging, or standard management alone?

#### 3.4.2. Recommendation 4

In adult patients with acute pancreatitis, the WSES panel suggests using serial procalcitonin measurements to support identification of infected pancreatic necrosis and guide antimicrobial decision-making as part of an integrated clinical, laboratory, organ-dysfunction, and imaging assessment.

Serial procalcitonin trends may be considered when infection is suspected, when antibiotic initiation or discontinuation is uncertain, or when the response to conservative or step-up management is being evaluated. [Certainty of evidence: Moderate. Strength of recommendation: Conditional, in favour.]

#### 3.4.3. Summary of Evidence

Acute pancreatitis has a broad clinical spectrum, ranging from mild self-limiting disease to necrotising pancreatitis with persistent organ failure. Infected pancreatic necrosis (IPN) is among its most serious complications and is associated with increased morbidity, intensive care admission, need for intervention, and mortality [50,51]. Timely recognition is clinically important because unnecessary antibiotic treatment of sterile necrosis exposes patients to avoidable harm, whereas delayed treatment of IPN may allow progression to sepsis and organ dysfunction.

Distinguishing sterile inflammation from infection remains challenging. Fever, leukocytosis, elevated C-reactive protein (CRP), systemic inflammatory response syndrome, and organ dysfunction may occur in severe acute pancreatitis without bacterial infection, while imaging findings can be indeterminate, particularly early in the disease course [52]. PCT has been extensively evaluated in this setting because elevated or persistently increasing concentrations are more closely associated with bacterial infection than with sterile pancreatic inflammation.

Available evidence supports the diagnostic value of PCT for IPN. Earlier prospective studies showed significantly higher PCT concentrations in patients who developed infected necrosis than in those with sterile necrosis [52,53,54]. A systematic review by Mofidi et al. reported a pooled sensitivity of 0.80 and specificity of 0.91 for predicting IPN, with an area under the receiver operating characteristic curve (AUC) of 0.91 [49]. A 2026 meta-analysis including 16 diagnostic studies, 2156 patients, and 512 cases of IPN confirmed good diagnostic performance, with a pooled sensitivity of 0.82, specificity of 0.84, and AUC of 0.90 [55]. Across studies, the most informative cut-offs generally ranged from approximately 1.0 to 3.5 ng/mL, although assay characteristics, sampling time, disease severity, and diagnostic definitions contributed to heterogeneity.

Timing and trajectory materially influence diagnostic performance. During the first 72 h, elevated PCT may reflect the intensity of the early inflammatory response and has limited ability to distinguish sterile from infected necrosis. A 2024 systematic review and meta-analysis reported an AUC of 0.69 for PCT measured within the first 72 h, increasing to 0.86 after 72 h when persistently elevated values were considered [55]. The 2026 meta-analysis similarly found that serial dynamic monitoring performed better than a single admission measurement, with an AUC of approximately 0.93 compared with 0.85 [54]. These findings support serial measurement when infection is suspected: persistently elevated or increasing values strengthen the suspicion of IPN or another septic complication, whereas low or decreasing values support continued observation when the clinical and radiological course is otherwise reassuring.

PCT also provides actionable evidence for antimicrobial stewardship in acute pancreatitis. In the PROCAP randomized trial, 260 patients were assigned to PCT-guided care or usual care. Antibiotics were prescribed during the index admission in 45% of patients in the PCT-guided group compared with 63% of those receiving usual care, with an adjusted risk difference of −15.6%. PCT guidance also reduced the mean duration of antibiotic exposure from 5.8 to 4.5 days without increasing clinical infections, hospital-acquired infections, adverse events, or mortality [56]. These findings support a reduction in antibiotic prescribing with a predefined PCT algorithm. No increase in adverse clinical outcomes was observed, although the trial does not establish equivalent safety across all patient groups.

Interpretation of PCT in acute pancreatitis requires consideration of timing and clinical context. Severe systemic inflammation, organ dysfunction, and the extent of pancreatic injury may cause transient elevations, while proposed thresholds vary according to sampling time, disease severity, assay characteristics, and treatment pathway. A single early elevation does not establish IPN and should also prompt consideration of alternative infectious sources, including cholangitis, pneumonia, bloodstream infection, urinary tract infection, or catheter-related infection. Serial trends are therefore more informative than isolated values and should be integrated with clinical evolution, organ-dysfunction scores such as APACHE II or SOFA, imaging findings, microbiology when available, and multidisciplinary expertise. Conversely, persistently low or decreasing PCT values may support avoidance or discontinuation of antibiotics when bacterial infection is not otherwise suspected.

When IPN is suspected or confirmed, management follows a multidisciplinary step-up strategy, beginning with appropriate antimicrobial therapy and progressing, when required, to percutaneous or endoscopic drainage and minimally invasive or open necrosectomy [57]. PCT can strengthen or reduce the suspicion of infection and support antimicrobial decisions during this pathway, but the need for and timing of drainage or necrosectomy should remain based on the patient’s clinical condition, imaging findings, anatomical characteristics, and response to initial treatment.

Overall, PCT provides clinically meaningful information in acute necrotising pancreatitis. Serial measurements improve identification of IPN compared with isolated early values, while PCT-guided algorithms reduce antibiotic exposure without increasing infection-related complications or harm. Its greatest value lies in combining dynamic biomarker information with clinical assessment, organ-dysfunction trends, microbiology, and imaging to recognize infection promptly and support proportionate antimicrobial management.

### 3.5. PCT Interpretation in Trauma Patients

#### 3.5.1. Key Clinical Question 5

In adult trauma patients, do serial PCT measurements help distinguish infectious complications from trauma-induced systemic inflammation compared with clinical assessment, conventional inflammatory markers, imaging, or standard ICU monitoring alone?

#### 3.5.2. Recommendation 5

In adult trauma patients, the WSES panel suggests serial procalcitonin monitoring to assist in distinguishing post-traumatic infectious complications from sterile injury-related systemic inflammatory response syndrome.

Persistent elevation beyond the early inflammatory phase, failure to decrease within 48–72 h, or a secondary rise should prompt reassessment for infectious complications, including pneumonia, catheter-related infection, wound infection, intra-abdominal infection, missed bowel injury, or other ICU-acquired infections. [Certainty of evidence: Low. Strength of recommendation: Conditional, in favour.]

#### 3.5.3. Summary of Evidence

Severe trauma commonly induces a systemic inflammatory response syndrome (SIRS) characterised by fever, tachycardia, leukocytosis, elevated C-reactive protein (CRP), cytokine release, complement activation, endothelial dysfunction, ischaemia–reperfusion injury, and immune dysregulation [58,59,60]. These changes may occur as part of the host response to tissue injury and repair, even in the absence of infection. Distinguishing sterile post-traumatic inflammation from early infectious complications is therefore particularly challenging in critically ill patients, those requiring emergency surgery, and patients admitted to the ICU.

PCT may also increase during the early post-traumatic phase in the absence of infection. During the first 24–48 h, PCT concentrations may reflect injury severity, haemorrhagic shock, tissue damage, ischaemia–reperfusion, emergency surgery, massive transfusion, or a high Injury Severity Score rather than bacterial infection [61,62,63,64,65,66]. Consequently, an isolated PCT measurement obtained immediately after trauma has limited diagnostic specificity and should be interpreted within the broader clinical and physiological context.

The clinical value of PCT in trauma patients derives primarily from its kinetics after this early inflammatory phase. Following an uncomplicated course, the initial trauma-induced elevation is expected to decrease. Persistent elevation beyond the early post-traumatic phase, failure to decline within 48–72 h, or a secondary rise after an initial decrease has been associated with infectious complications, sepsis, organ dysfunction, and an unfavourable clinical course [62,63]. Serial PCT trends may therefore identify patients requiring focused reassessment for an occult or evolving infection, while a declining trajectory is generally consistent with resolution of the initial injury-related inflammatory response.

Evidence from trauma ICU populations supports this association. In a retrospective study of 149 trauma ICU patients, Ramasawmy et al. reported significantly higher median PCT concentrations in patients with positive blood cultures than in those without bacteraemia (30.5 vs. 6.6 ng/mL; *p* = 0.002). A threshold above 2.0 ng/mL showed high sensitivity but poor specificity, whereas a threshold above 31.0 ng/mL derived from this trauma ICU population improved specificity at the expense of sensitivity [65]. These findings support the contribution of PCT to the recognition of bacteraemia in trauma patients but also demonstrate that absolute thresholds derived from individual populations should not be applied as universal decision limits.

The clinical relevance of serial PCT monitoring extends to the range of post-traumatic and ICU-acquired infections that may develop during the subsequent course, including pneumonia or ventilator-associated pneumonia, catheter-related bloodstream infection, wound or surgical-site infection, intra-abdominal infection, undrained collections, and delayed recognition of hollow-viscus injury. Persistent or increasing PCT values are particularly concerning when accompanied by fever, new or worsening organ dysfunction, haemodynamic instability, respiratory deterioration, or abdominal findings.

Compared with CRP and white blood cell count, the more rapid kinetics of PCT may provide additional information for identifying an evolving bacterial infection. CRP and leukocytosis are frequently elevated after trauma and may remain abnormal because of tissue injury, operative stress, and sterile systemic inflammation. Evidence also supports a multimarker approach: in a prospective study of 100 patients with severe trauma, a model combining PCT, CRP, and serum amyloid A achieved an area under the receiver operating characteristic curve of 0.89 for predicting sepsis [66]. PCT should therefore complement clinical assessment and conventional biomarkers rather than replace them. Its interpretation should also account for haemorrhagic shock, transfusion, repeated surgery, renal dysfunction, and other factors associated with critical illness.

In selected patients admitted to the ICU after major trauma, a practical strategy may include baseline PCT measurement at admission, followed by serial measurements during the first 48–72 h and continued monitoring in high-risk patients or when clinical deterioration occurs. Persistent elevation beyond the early post-traumatic phase, failure to decrease within 48–72 h, or a secondary rise after an initial decline should prompt integrated reassessment for infection. This should include repeat clinical examination, review of organ-dysfunction trends, appropriate microbiological cultures, and evaluation of central lines, wounds, and drains. Chest imaging should be obtained when pneumonia is suspected, and abdominal CT should be considered when intra-abdominal infection, missed hollow-viscus injury, or an undrained collection is clinically suspected.

Overall, the available evidence supports serial PCT monitoring as a clinically useful adjunct for the early recognition of septic complications after trauma and for distinguishing evolving infection from sterile injury-related inflammation. Its principal value lies in the trajectory beyond the early post-traumatic phase rather than in an isolated value or universal cut-off. Although the evidence is predominantly observational and affected by trauma severity, shock, surgical procedures, and ICU-related factors, serial PCT trends can identify an unfavourable course and prompt timely, targeted investigation within an integrated trauma and critical-care assessment pathway.

### 3.6. PCT for Early Detection or Exclusion of Postoperative Anastomotic Leak

#### 3.6.1. Key Clinical Question 6

In adult patients undergoing gastrointestinal or colorectal surgery with an anastomosis, does postoperative PCT measurement improve early detection or exclusion of anastomotic leak and intra-abdominal infection compared with clinical assessment, C-reactive protein, white blood cell count, imaging, or standard postoperative monitoring alone?

#### 3.6.2. Recommendation 6

In adult patients undergoing gastrointestinal or colorectal surgery with an anastomosis, the WSES panel suggests postoperative procalcitonin monitoring as part of early risk stratification for anastomotic leak and postoperative intra-abdominal infection.

Low and decreasing procalcitonin values may support a low probability of anastomotic leak in clinically improving patients, whereas persistently elevated values or a secondary rise should prompt reassessment and imaging when clinically indicated. [Certainty of evidence: Low. Strength of recommendation: Conditional, in favour.]

#### 3.6.3. Summary of Evidence

Anastomotic leak is one of the most serious complications after gastrointestinal and colorectal surgery. It results from impaired healing or disruption of the anastomosis and may be favoured by bowel ischaemia, tension, technical factors, contamination, malnutrition, corticosteroid therapy, poor tissue perfusion, and respiratory or systemic comorbidity. Anastomotic leak is associated with prolonged hospital stay, antibiotic therapy, percutaneous drainage or reoperation, stoma formation, increased morbidity and mortality, and, in oncological surgery, worse long-term outcomes.

Early diagnosis is essential, but the clinical presentation may be subtle, particularly in older, immunocompromised, or critically ill patients. Initial manifestations may include fever, tachycardia, ileus, abdominal pain, delayed gastrointestinal recovery, or non-mechanical bowel obstruction. When leakage is contained, the clinical picture may be limited to an abscess or localised intra-abdominal infection; when contamination is diffuse, generalised peritonitis and septic shock may develop [67,68].

Conventional postoperative inflammatory markers are useful but have important limitations. CRP and white blood cell count may increase as part of the expected inflammatory response to surgical trauma and may remain elevated even after uncomplicated recovery. PCT is more closely associated with bacterial infection and the endotoxin-mediated systemic response. After surgery, a transient PCT increase may occur, but concentrations are expected to decrease when there is no ongoing bacterial contamination or infectious complication [24,69,70,71]. Postoperative trends are therefore generally more informative than isolated values.

Several studies have evaluated PCT as an early biomarker of postoperative intra-abdominal infection and anastomotic leak after gastrointestinal surgery, particularly after colorectal procedures [72,73,74,75,76,77,78,79]. PCT measured during postoperative days 3–5 has shown diagnostic value for leak detection and postoperative infectious complications. Its additional value beyond CRP and standard clinical assessment, however, remains incompletely established. Cousin et al. [74] found no significant difference between CRP and PCT in diagnostic accuracy for postoperative intra-abdominal infection after elective colorectal surgery. In the PREDICS study [71], combined PCT and CRP measurements on postoperative day 5 achieved an AUC of 0.901 compared with 0.806 for CRP alone, corresponding to an absolute difference of 0.095 calculated from the reported values. These findings suggest potential complementary diagnostic information, although improved discrimination does not itself establish better clinical decisions or outcomes.

Low PCT values, commonly below approximately 0.5–1.0 ng/mL, may have useful negative predictive value, especially when accompanied by a decreasing trend and clinical improvement. Negative predictive value also depends on the prevalence of anastomotic leak in the population assessed. In patients managed within enhanced recovery pathways, reassuring PCT trends may contribute to identifying patients at low risk of occult leak and support decisions regarding early discharge or the need for imaging, alongside CRP and standard clinical assessment. A reassuring PCT result should not preclude imaging when clinical suspicion persists.

Conversely, persistently elevated PCT concentrations or a secondary rise after an initial decline may suggest intra-abdominal infection, anastomotic leak, abscess, or evolving sepsis. PCT values above approximately 1–2 ng/mL after postoperative day 3 have been associated with a higher probability of leak or postoperative intra-abdominal infection, particularly when accompanied by tachycardia, ileus, abdominal pain, fever, or delayed recovery [70]. However, proposed thresholds vary according to the timing of measurement, operative procedure, patient population, renal function, and definition or severity of anastomotic leak. They should therefore be considered indicative rather than definitive and should not be applied without clinical correlation.

Recent evidence indicates that PCT diagnostic performance may be lower in specific populations. In older adults undergoing colorectal cancer surgery, Ramírez-Giraldo et al. reported poor overall discrimination of a single PCT measurement on postoperative day 3 for anastomotic leak, with an area under the receiver operating characteristic curve of 0.68. Diagnostic performance improved when patients with ileostomy were excluded and when the analysis was restricted to grade C leaks [77]. Similarly, a recent systematic review and meta-analysis found higher postoperative PCT concentrations in patients with colorectal anastomotic leak. In analyses restricted to patients with colorectal cancer, the pooled differences remained statistically significant on postoperative days 2 and 3, but lost statistical significance in leave-one-out sensitivity analyses [78]. These findings do not negate the potential value of serial PCT monitoring, but they caution against relying on a single postoperative PCT measurement to confirm or exclude anastomotic leak in older or oncological patients.

PCT and CRP may provide complementary information. CRP is widely used and has established value in postoperative monitoring but lacks specificity for bacterial infection. PCT may improve risk discrimination when combined with CRP, white blood cell count, clinical examination, and the postoperative recovery trajectory [69,70]. This combined interpretation may be particularly useful in enhanced recovery pathways, in which early discharge requires reliable identification of patients at low risk of occult leak. Nevertheless, PCT should support rather than independently determine discharge or imaging decisions.

When PCT remains elevated or rises after an initial decline, the finding should prompt careful reassessment, including clinical examination, review of postoperative recovery and organ-dysfunction trends, evaluation of other biomarkers, and CT imaging when clinically indicated. Conversely, low and decreasing PCT values may contribute to assessment of leak risk in a clinically improving patient. Whether incorporating PCT into assessment alongside clinical findings and CRP can safely reduce imaging, shorten observation, or facilitate earlier discharge has not been established.

A practical monitoring approach may include an early postoperative measurement, followed by reassessment around postoperative day 3 and, when clinically indicated, additional measurements during postoperative days 4–5, consistent with protocols evaluated in the literature [63,72,73,74,75,76,77,78,79]. Values below the lower thresholds studied, particularly when decreasing and accompanied by clinical improvement, provide reassuring evidence against an evolving leak. Values exceeding the higher ranges reported in the literature, or a secondary rise, should increase suspicion and support further investigation, whereas intermediate values require interpretation within the overall clinical trajectory. Patient-specific factors, including renal dysfunction, immunosuppression, major surgical stress, trauma, and oncological disease, should also be considered because they may alter baseline concentrations or postoperative kinetics. When postoperative antibiotics have been initiated but no leak or other infection is identified, low and decreasing PCT values may support avoidance of unnecessary prolonged treatment or earlier discontinuation within an antimicrobial stewardship pathway [35,36,37].

Overall, postoperative PCT monitoring may support early risk stratification and contribute to the detection or exclusion of anastomotic leak and postoperative intra-abdominal infection in selected patients undergoing gastrointestinal or colorectal surgery. Its greatest value appears to lie in serial trends during postoperative days 3–5 and in their integration with clinical assessment, CRP, white blood cell count, imaging, and enhanced recovery criteria. Low and decreasing values may provide clinically useful reassurance, whereas persistent elevation or a secondary rise should prompt timely reassessment for an occult leak or other postoperative infectious complication.

### 3.7. Practical Considerations and Research Priorities

The 2021 surgical-pathway consensus by Sartelli et al. addressed antibiotic duration and relaparotomy, but subsequent evidence illustrates the difficulty of translating PCT results into consistent decisions across different abdominal surgical settings. Jerome et al. found only moderate diagnostic accuracy for postoperative infection after major gastrointestinal surgery, while Perrella et al. reported that CRP, but not PCT, reflected antibiotic response in their cohort after major abdominal surgery. Differences in patient populations, clinical questions, measurement times, and proposed thresholds leave surgeons without sufficiently specific guidance on how to incorporate PCT into routine assessment and action [26,42,79].

Across the clinical scenarios addressed in this position paper, PCT may support surgical decision-making when interpreted according to the timing of measurement, the threshold ranges evaluated in the relevant clinical setting, the direction of change, and the patient’s overall trajectory. Values below the lower thresholds studied for a specific setting, particularly when decreasing and accompanied by clinical improvement, may support a lower probability of ongoing bacterial infection or postoperative infectious complications. After adequate source control, a low PCT concentration, commonly below 0.5–1.0 ng/mL depending on the protocol used, or a substantial decline from peak values, commonly at least 80%, may support early antibiotic discontinuation when treatment duration remains uncertain. These criteria have been evaluated in surgical and general ICU algorithms to reduce antibiotic exposure, although their applicability and safety should be interpreted in the context of the population and protocol studied [30,32,35,36,37].

Conversely, values above the upper ranges reported in the literature, persistent elevation, failure to decline, or a secondary rise increase concern for ongoing infection, inadequate source control, or an unfavourable postoperative course and should prompt targeted reassessment. Such findings do not independently diagnose infection or identify its source. Values within intermediate ranges should be interpreted primarily through serial kinetics and integrated with clinical, laboratory, microbiological, and imaging findings.

Interpretation should also account for patient-specific factors, including renal dysfunction, immunosuppression, major trauma or surgery, and oncological disease, which may alter baseline PCT concentrations or postoperative kinetics.

Major surgery, severe tissue injury, and shock may increase PCT in the absence of bacterial infection [80]. Following liver surgery, elevations may also reflect surgery-related hepatic injury and perioperative inflammatory responses, although the underlying mechanisms are not fully established [81]. Renal dysfunction may be associated with elevated baseline concentrations and altered kinetics, while renal replacement therapy can modify PCT removal depending on the modality and membrane characteristics [80,82]. Ongoing production may offset extracorporeal removal, so changes during treatment cannot be attributed solely to infection resolution or persistence [82]. Immunosuppression and oncological disease may further complicate interpretation according to the underlying condition and treatment [80].

Analytical variability also warrants consideration. Differences between PCT assays can affect results near clinical decision thresholds, particularly at low concentrations; serial measurements should therefore preferably use the same assay platform [80,83]. Absolute thresholds may be less transferable across populations and laboratories, making within-patient trends potentially more informative in some settings. However, relative changes are also influenced by the baseline or peak selected, sampling intervals, evolving organ dysfunction, and concurrent treatments [80,82]. Neither approach eliminates the need for clinical assessment.

**Figure 1:**
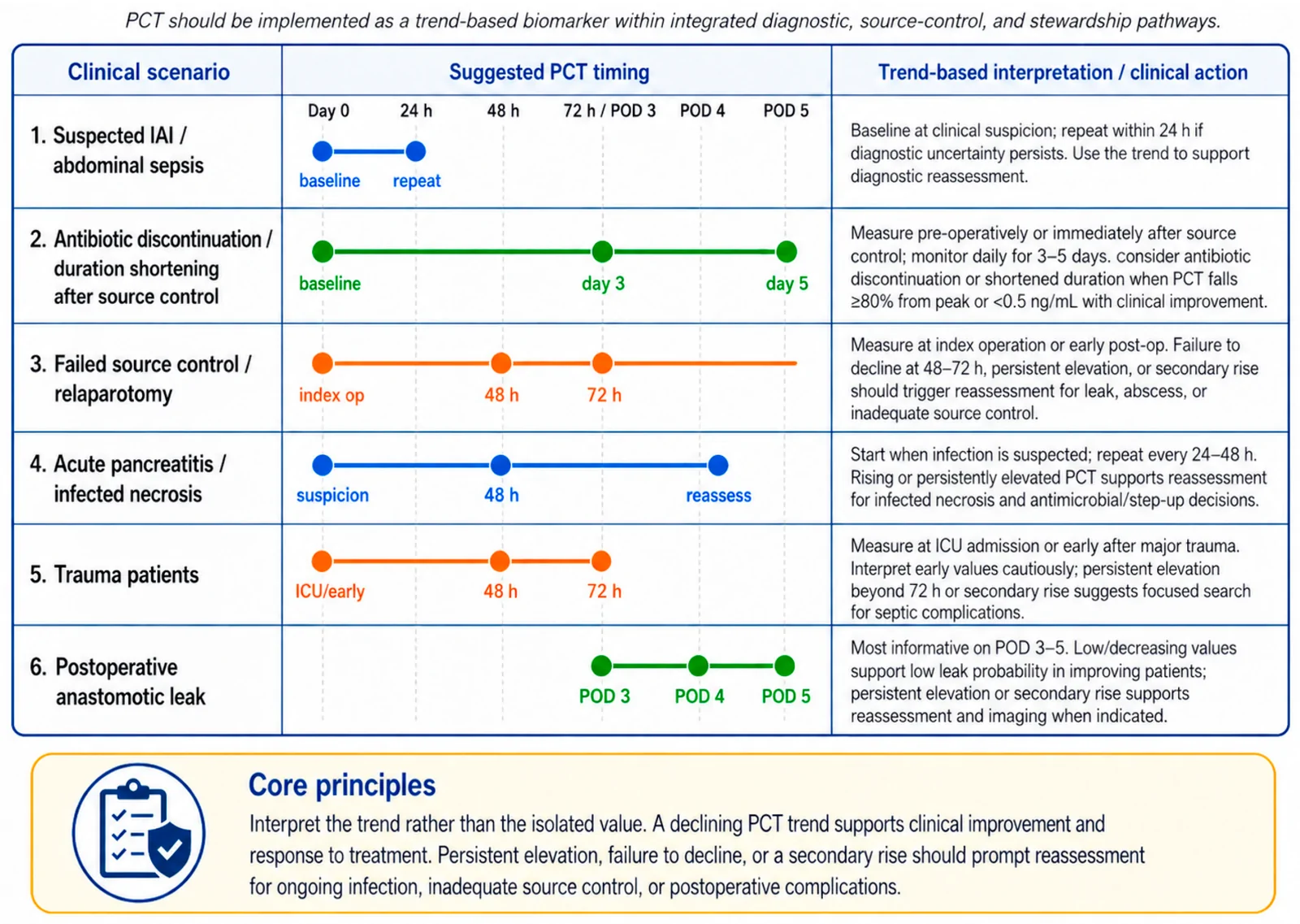
Suggested timing of serial procalcitonin assessment across abdominal and emergency surgical scenarios. Suggested schedules apply to selected patients when PCT measurement addresses a defined clinical uncertainty and may influence management; routine serial testing is not implied.

**Figure 2:**
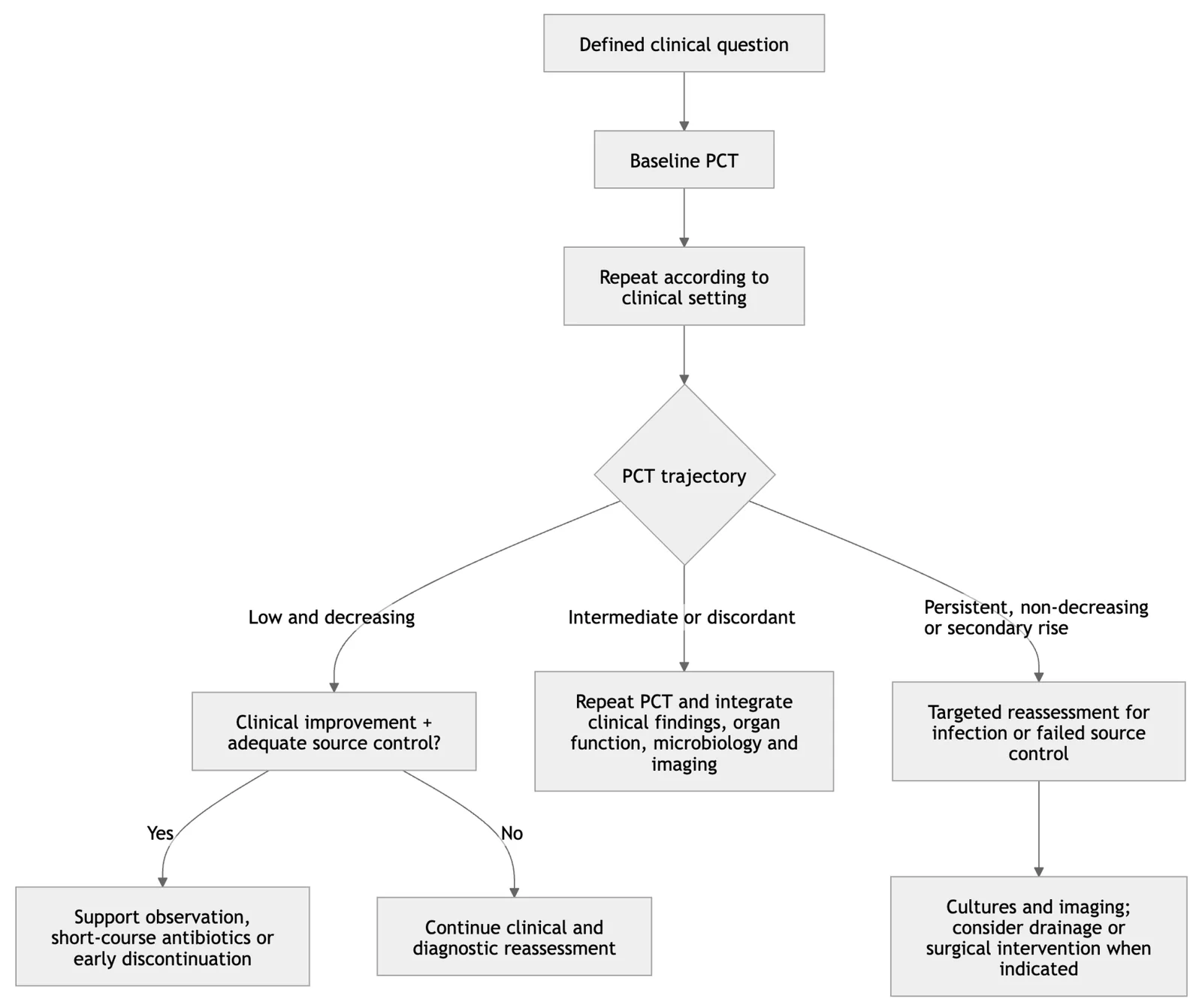
Practical flowchart for the interpretation of serial procalcitonin measurements in abdominal and emergency surgery.

There is no single measurement schedule suitable for all six scenarios. A practical approach may include an initial measurement at presentation, ICU admission, source-control intervention, or during the immediate postoperative period, followed by reassessment within 24–48 h and further measurements according to the clinical scenario and the protocols evaluated in the literature. Assessment for anastomotic leak is generally most informative during postoperative days 3–5. Suggested timing for baseline and serial PCT measurements across the clinical scenarios addressed in this position paper is summarised in Figure 1. A practical approach to the interpretation of serial PCT trajectories and the corresponding clinical reassessment is presented in Figure 2.

Reported cut-offs should support, rather than independently determine, decisions regarding antimicrobial therapy, imaging, drainage, reintervention, or discharge. Serial PCT may serve as an additional warning signal prompting reassessment for infection or source-control failure. However, the evidence reviewed does not establish whether PCT changes consistently precede clinically apparent deterioration or whether acting on these changes improves outcomes.

Future prospective studies should validate scenario-specific thresholds and measurement schedules, clarify the timing of PCT changes relative to clinical deterioration, and determine whether PCT-informed pathways improve patient outcomes, reduce antimicrobial exposure and unnecessary investigations or interventions, and offer cost-effective use of healthcare resources.

### 3.8. Limitations

This position paper is based on a focused literature review and expert consensus. No systematic review or formal study-level risk-of-bias assessment was conducted. The supporting evidence is heterogeneous and partly derived from mixed ICU populations or elective surgical cohorts, limiting its direct applicability to emergency general surgery. The incremental benefit of PCT beyond established biomarkers and clinical assessment remains uncertain in some domains. The recommendations should therefore be interpreted within their clinical context and refined as further evidence becomes available.

## 4. Conclusions

This WSES position paper highlights the potential role of PCT in some of the most difficult decisions faced by abdominal and emergency surgeons. PCT does not provide answers on its own, but serial measurements can add useful information when the clinical course is unclear and conventional inflammatory markers are difficult to interpret.

Used alongside clinical assessment, PCT may help surgeons limit unnecessary antibiotic exposure, recognise an unfavourable course potentially earlier, and identify patients who need further investigation or intervention. This approach may support more timely and individualised care. However, universal thresholds cannot yet be recommended, and prospective studies are needed to define how PCT should best be used in each clinical setting.

## Figures and Tables

**Table 1:** PICO questions addressed in the WSES position paper on procalcitonin-guided decision-making in abdominal and emergency surgery.

	Clinical Domain	PICO Question
1	Diagnosis of intra-abdominal infection	In adult patients with suspected intra-abdominal infection or abdominal sepsis, does measurement of PCT, alone or as part of serial biomarker assessment, improve diagnostic accuracy or clinical decision-making compared with clinical assessment, conventional laboratory markers, microbiology, and imaging alone?
2	Antibiotic discontinuation after source control	In adult patients receiving antimicrobial treatment for intra-abdominal infection after adequate source control, does PCT-guided antibiotic discontinuation reduce antibiotic exposure compared with fixed-duration or clinically guided therapy, without increasing treatment failure or infectious recurrence?
3	Suspected failed source control	In adult patients following surgery for secondary peritonitis or abdominal sepsis, do serial PCT trends help identify failed source control or the need for repeat intervention compared with clinical assessment, conventional inflammatory markers, imaging, or standard postoperative monitoring alone?
4	Acute pancreatitis and infected pancreatic necrosis	In adult patients with acute pancreatitis, do PCT measurements improve identification of infected pancreatic necrosis and support antimicrobial or interventional management compared with clinical assessment, conventional biomarkers, imaging, or standard management alone?
5	Infectious complications after trauma	In adult trauma patients, do serial PCT measurements help distinguish infectious complications from trauma-induced systemic inflammation compared with clinical assessment, conventional inflammatory markers, imaging, or standard ICU monitoring alone?
6	Postoperative anastomotic leak	In adult patients undergoing gastrointestinal or colorectal surgery with an anastomosis, does postoperative PCT measurement improve early detection or exclusion of anastomotic leak and intra-abdominal infection compared with clinical assessment, CRP, WBC count, imaging, or standard postoperative monitoring alone?

Abbreviations: CRP, C-reactive protein; ICU, intensive care unit; PCT, procalcitonin; PICO, Population, Intervention or Index test, Comparator, and Outcome; WBC, white blood cell.

**Table 2:** Summary of the WSES recommendations on procalcitonin-informed decision-making in abdominal and emergency surgery.

	Clinical Domain	WSES Recommendation	Certainty of Evidence	Strength and Direction
1	Diagnosis of intra-abdominal infection	In adult patients with suspected intra-abdominal infection or abdominal sepsis and persistent diagnostic uncertainty, particularly those at risk of complicated or systemic infection, the WSES panel suggests using PCT as an adjunct to integrated clinical, laboratory, microbiological, and imaging assessment. Serial measurements may support reassessment in selected postoperative or critically ill patients. PCT should not be used as a stand-alone diagnostic test or delay imaging, empirical antimicrobial treatment in unstable patients, or urgent source control.	Low	Conditional, in favour
2	Antibiotic discontinuation after source control	In adult patients treated for intra-abdominal infection after adequate source control, the WSES panel suggests using serial PCT trends, together with clinical assessment, to support antibiotic discontinuation within an antimicrobial stewardship pathway. A substantial decline from peak PCT concentrations, commonly ≥80%, or a low absolute value may support discontinuation in clinically improving patients, according to locally validated protocols and assay availability.	Moderate	Conditional, in favour
3	Suspected failed source control	In adult patients with suspected tertiary peritonitis or persistent or recurrent abdominal sepsis following initial source control, the WSES panel suggests incorporating serial PCT measurements into the clinical reassessment pathway to support early identification of failed source control and timely evaluation for repeat intervention. Persistent elevation, failure to decline within 48–72 h, or a secondary rise should prompt targeted reassessment, including imaging and consideration of additional source-control procedures when clinically indicated. PCT kinetics should support, but not independently determine, repeat source-control decisions.	Low	Conditional, in favour
4	Acute pancreatitis and infected pancreatic necrosis	In adult patients with acute pancreatitis, the WSES panel suggests using serial PCT measurements to support identification of infected pancreatic necrosis and antimicrobial decision-making as part of integrated clinical, laboratory, organ-dysfunction, and imaging assessment. Persistent elevation or an increase beyond 72 h strengthens suspicion of infection. PCT should not independently determine the need or timing of drainage or necrosectomy.	Moderate	Conditional, in favour
5	Infectious complications after trauma	In adult trauma patients, the WSES panel suggests serial PCT monitoring to assist in distinguishing post-traumatic infectious complications from sterile injury-related systemic inflammation. An early value may provide a useful baseline; failure to decline within 48–72 h or a secondary rise should prompt investigation for infection.	Low	Conditional, in favour
6	Postoperative anastomotic leak	In adult patients undergoing gastrointestinal or colorectal surgery with an anastomosis, the WSES panel suggests postoperative PCT monitoring as part of early risk stratification for anastomotic leak and postoperative intra-abdominal infection. Low and decreasing values may support a low probability of leak in clinically improving patients, whereas persistent elevation or a secondary rise should prompt reassessment and imaging when clinically indicated.	Low	Conditional, in favour

PCT should support, but not independently determine, diagnostic, antimicrobial, imaging, drainage, repeat source-control, or discharge decisions. Abbreviations: PCT, procalcitonin; WSES, World Society of Emergency Surgery.

## Data Availability

The original contributions presented in this study are included in the article. Further inquiries can be directed to the corresponding author.

## Supplementary Materials

The following supporting information can be downloaded at: https://www.mdpi.com/article/10.3390/antibiotics15101042/s1. Table S1: Recommendation 1 Diagnosis and reassessment of intra-abdominal infection and selected evidence; Table S2: Recommendation 2 Antibiotic discontinuation after adequate source control; selected evidence; Table S3: Recommendation 3 Reassessment for suspected source-control failure; selected evidence; Table S4: Recommendation 4 Acute pancreatitis and infected pancreatic necrosis; selected evidence; Table S5: Recommendation 5 Infectious complications after trauma; selected evidence; Table S6: Recommendation 6 Postoperative anastomotic leak; selected evidence.

## References

[1] Becker K.L., Nylén E.S., White J.C., Müller B., Snider R.H. (2004). Procalcitonin and the calcitonin gene family of peptides in inflammation, infection, and sepsis: A journey from calcitonin back to its precursors. J. Clin. Endocrinol. Metab..

[2] Le Moullec J.M., Jullienne A., Chenais J., Lasmoles F., Guliana J.M., Milhaud G., Moukhtar M.S. (1984). The complete sequence of human preprocalcitonin. FEBS Lett..

[3] Cleland D.A., Eranki A.P. Procalcitonin. StatPearls.

[4] Assicot M., Gendrel D., Carsin H., Raymond J., Guilbaud J., Bohuon C. (1993). High serum procalcitonin concentrations in patients with sepsis and infection. Lancet.

[5] Christ-Crain M., Müller B. (2005). Procalcitonin in bacterial infections--hype, hope, more or less?. Swiss Med. Wkly..

[6] Schuetz P., Albrich W., Mueller B. (2011). Procalcitonin for diagnosis of infection and guide to antibiotic decisions: Past, present and future. BMC Med..

[7] Xu Q., Qian Y., Guan M., Liu M., Wei S., Cao Y., Jiang X., Lu W., Zha L. (2026). Trends in mortality, pathogen distribution, and antimicrobial resistance of peritoneal and intra-abdominal infections from 1990 to 2021: A cross-sectional analysis of the MICROBE database. Int. J. Surg..

[8] Brogi E., Cremonini C., Ceresoli M., Catena F., Gurrado A., Forfori F., Ghiadoni L., Melai E., Sartelli M., Coccolini F. (2026). IRIS Study Group. Epidemiology and outcome of intra-abdominal infections in intensive care unit in Italy from the Italian Register of complicated Intra-abdominal InfectionS—The IRIS study: A prospective observational nationwide study. J. Anesth. Analg. Crit. Care.

[9] Tian B.W.C.A., Agnoletti V., Ansaloni L., Coccolini F., Bravi F., Sartelli M., Vallicelli C., Catena F. (2023). Management of intra-abdominal infections: The role of procalcitonin. Antibiotics.

[10] Khilnani G.C., Tiwari P., Zirpe K.G., Chaudhry D., Govil D., Dixit S., Kulkarni A.P., Todi S.K., Hadda V., Jain N. (2022). Guidelines for the use of procalcitonin for rational use of antibiotics. Indian J. Crit. Care Med..

[11] Richardson W.S., Wilson M.C., Nishikawa J., Hayward R.S.A. (1995). The well-built clinical question: A key to evidence-based decisions. ACP J. Club.

[12] Page M.J., McKenzie J.E., Bossuyt P.M., Boutron I., Hoffmann T.C., Mulrow C.D., Shamseer L., Tetzlaff J.M., Akl E.A., Brennan S.E. (2021). The PRISMA 2020 statement: An updated guideline for reporting systematic reviews. BMJ.

[13] Balshem H., Helfand M., Schünemann H.J., Oxman A.D., Kunz R., Brozek J., Vist G.E., Falck-Ytter Y., Meerpohl J., Norris S. (2011). GRADE guidelines: 3. Rating the quality of evidence. J. Clin. Epidemiol..

[14] Kim J.H. (2022). Clinical utility of procalcitonin on antibiotic stewardship: A narrative review. Infect. Chemother..

[15] Pepys M.B., Hirschfield G.M. (2003). C-reactive protein: A critical update. J. Clin. Investig..

[16] Simon L., Gauvin F., Amre D.K., Saint-Louis P., Lacroix J. (2004). Serum procalcitonin and C-reactive protein levels as markers of bacterial infection: A systematic review and meta-analysis. Clin. Infect. Dis..

[17] Ozen C., Karasoy D., Yalcinkaya A., Pedersen S.H., Fagerberg S.K., Hindersson P., Leutscher P.D.C., Holte K. (2025). Evaluation of procalcitonin versus conventional inflammatory biomarkers for clinical severity grading in patients with intra-abdominal infection. Langenbecks Arch. Surg..

[18] Covino M., Fransvea P., Rosa F., Cozza V., Quero G., Simeoni B., Gasbarrini A., Alfieri S., Franceschi F., Sganga G. (2021). Early procalcitonin assessment in the emergency department in patients with intra-abdominal infection: An excess or a need?. Surg. Infect..

[19] Yu C.W., Juan L.I., Wu M.H., Shen C.J., Wu J.Y., Lee C.C. (2013). Systematic review and meta-analysis of the diagnostic accuracy of procalcitonin, C-reactive protein and white blood cell count for suspected acute appendicitis. Br. J. Surg..

[20] Dale L. (2022). The use of procalcitonin in the diagnosis of acute appendicitis: A systematic review. Cureus.

[21] Jeger V., Pop R., Forudastan F., Barras J.P., Zuber M., Piso R.J. (2017). Is there a role for procalcitonin in differentiating uncomplicated and complicated diverticulitis in order to reduce antibiotic therapy? A prospective diagnostic cohort study. Swiss Med. Wkly..

[22] Schena C.A., Covino M., Laterza V., Quero G., La Greca A., Cina C., De’aNgelis N., Marchegiani F., Sganga G., Gasbarrini A. (2024). The role of procalcitonin as a risk stratification tool of severity, prognosis, and need for surgery in patients with acute left-sided colonic diverticulitis. Surgery.

[23] Yaow C.Y.L., Chong R.I.H., Chan K.S., Chia C.T.W., Shelat V.G. (2023). Should procalcitonin be included in acute cholecystitis guidelines? A systematic review. Medicina.

[24] Arbutina D.D., Milic L., Cuk V.V., Juloski J.T., Radulovic R., Starcevic A., Karamarkovic A.R. (2022). Significance of biomarkers in early diagnosis of abdominal sepsis. Chirurgia.

[25] Yang S.K., Xiao L., Zhang H., Xu X.X., Song P.A., Liu F.Y., Sun L. (2014). Significance of serum procalcitonin as biomarker for detection of bacterial peritonitis: A systematic review and meta-analysis. BMC Infect. Dis..

[26] Perrella A., Giuliani A., De Palma M., Castriconi M., Molino C., Vennarecci G., Antropoli C., Esposito C., Calise F., Frangiosa A. (2022). C-reactive protein but not procalcitonin may predict antibiotic response and outcome in infections following major abdominal surgery. Updat. Surg..

[27] Sartelli M., Barie P.S., Agnoletti V., Al-Hasan M.N., Ansaloni L., Biffl W., Buonomo L., Blot S., Cheadle W.G., Coimbra R. (2024). Intra-abdominal infections survival guide: A position statement by the Global Alliance For Infections In Surgery. World J. Emerg. Surg..

[28] Sawyer R.G., Claridge J.A., Nathens A.B., Rotstein O.D., Duane T.M., Evans H.L., Cook C.H., O’neill P.J., Mazuski J.E., Askari R. (2015). Trial of short-course antimicrobial therapy for intra-abdominal infection. N. Engl. J. Med..

[29] de Jong E., van Oers J.A., Beishuizen A., Vos P., Vermeijden W.J., Haas L.E., Loef B.G., Dormans T., van Melsen G.C., Kluiters Y.C. (2016). Efficacy and safety of procalcitonin guidance in reducing the duration of antibiotic treatment in critically ill patients: A randomised, controlled, open-label trial. Lancet Infect. Dis..

[30] Wacker C., Prkno A., Brunkhorst F.M., Schlattmann P. (2013). Procalcitonin as a diagnostic marker for sepsis: A systematic review and meta-analysis. Lancet Infect. Dis..

[31] Wirz Y., Meier M.A., Bouadma L., Luyt C.E., Wolff M., Chastre J., Tubach F., Schroeder S., Nobre V., Annane D. (2018). Effect of procalcitonin-guided antibiotic treatment on clinical outcomes in intensive care unit patients with infection and sepsis: A patient-level meta-analysis of randomized trials. Crit. Care.

[32] Pepper D.J., Sun J., Rhee C., Welsh J., Powers J.H., Danner R.L., Kadri S.S. (2019). Procalcitonin-Guided Antibiotic Discontinuation and Mortality in Critically Ill Adults: A Systematic Review and Meta-analysis. Chest.

[33] Papp M., Kiss N., Baka M., Trásy D., Zubek L., Fehérvári P., Harnos A., Turan C., Hegyi P., Molnár Z. (2023). Procalcitonin-guided antibiotic therapy may shorten length of treatment and may improve survival-a systematic review and meta-analysis. Crit. Care.

[34] Huang T.S., Huang S.S., Shyu Y.C., Lee C.H., Jwo S.C., Chen P.J., Chen H.Y. (2014). A procalcitonin-based algorithm to guide antibiotic therapy in secondary peritonitis following emergency surgery: A prospective study with propensity score matching analysis. PLoS ONE.

[35] Maseda E., Suarez-de-la-Rica A., Anillo V., Tamayo E., García-Bernedo C.A., Ramasco F., Villagran M.-J., Maggi G., Gimenez M.-J., Aguilar L. (2015). Procalcitonin-guided therapy may reduce length of antibiotic treatment in intensive care unit patients with secondary peritonitis: A multicenter retrospective study. J. Crit. Care.

[36] Slieker J.C., Aellen S., Eggimann P., Guarnero V., Schäfer M., Demartines N. (2017). Procalcitonin-guided antibiotics after surgery for peritonitis: A randomized controlled study. Gastroenterol. Res. Pract..

[37] Davies J. (2015). Procalcitonin. J. Clin. Pathol..

[38] Sartelli M., Chichom-Mefire A., Labricciosa F.M., Hardcastle T., Abu-Zidan F.M., Adesunkanmi A.K., Ansaloni L., Bala M., Balogh Z.J., Beltrán M.A. (2017). The management of intra-abdominal infections from a global perspective: 2017 WSES guidelines for management of intra-abdominal infections. World J. Emerg. Surg..

[39] van Ruler O., Mahler C.W., Boer K.R., Reuland E.A., Gooszen H.G., Opmeer B.C., de Graaf P.W., Lamme B., Gerhards M.F., Steller E.P. (2007). Comparison of on-demand vs planned relaparotomy strategy in patients with severe peritonitis: A randomized trial. JAMA.

[40] Hotchkiss R.S., Moldawer L.L., Opal S.M., Reinhart K., Turnbull I.R., Vincent J.L. (2016). Sepsis and septic shock. Nat. Rev. Dis. Primers.

[41] Assfalg V., Wolf P., Reim D., Hüser N., Hellbrügge G., Matevossian E., Friess H., Holzmann B., Emmanuel K.L., Novotny A.R. (2016). Procalcitonin ratio and on-demand relaparotomy for septic peritonitis: Validation of the focus index. Surg. Today.

[42] Sartelli M., Ansaloni L., Bartoletti M., Catena F., Cardi M., Cortese F., Di Marzo F., Pea F., Plebani M., Rossolini G.M. (2021). The role of procalcitonin in reducing antibiotics across the surgical pathway. World J. Emerg. Surg..

[43] Novotny A.R., Emmanuel K., Hueser N., Knebel C., Kriner M., Ulm K., Bartels H., Siewert J.R., Holzmann B. (2009). Procalcitonin ratio indicates successful surgical treatment of abdominal sepsis. Surgery.

[44] Jung B., Molinari N., Nasri M., Hajjej Z., Chanques G., Jean-Pierre H., Panaro F., Jaber S. (2013). Procalcitonin biomarker kinetics fails to predict treatment response in perioperative abdominal infection with septic shock. Crit. Care.

[45] Selberg O., Hecker H., Martin M., Klos A., Bautsch W., Köhl J. (2000). Discrimination of sepsis and systemic inflammatory response syndrome by determination of circulating plasma concentrations of procalcitonin, protein complement 3a, and interleukin-6. Crit. Care Med..

[46] Parli S.E., Trivedi G., Woodworth A., Chang P.K. (2018). Procalcitonin: Usefulness in acute care surgery and trauma. Surg. Infect..

[47] Coccolini F., Sartelli M., Sawyer R., Rasa K., Viaggi B., Abu-Zidan F., Soreide K., Hardcastle T., Gupta D., Bendinelli C. (2023). Source control in emergency general surgery: WSES, GAIS, SIS-E, SIS-A guidelines. World J. Emerg. Surg..

[48] De Waele J.J. (2024). Importance of timely and adequate source control in sepsis and septic shock. J. Intensive Med..

[49] Mofidi R., Suttie S.A., Patil P.V., Ogston S., Parks R.W. (2009). The value of procalcitonin at predicting the severity of acute pancreatitis and development of infected pancreatic necrosis: Systematic review. Surgery.

[50] Banks P.A., Bollen T.L., Dervenis C., Gooszen H.G., Johnson C.D., Sarr M.G., Tsiotos G.G., Vege S.S. (2013). Acute Pancreatitis Classification Working Group. Classification of acute pancreatitis—2012: Revision of the Atlanta classification and definitions by international consensus. Gut.

[51] Rau B., Steinbach G., Baumgart K., Gansauge F., Grünert A., Beger H.G. (2000). The clinical value of procalcitonin in the prediction of infected necrosis in acute pancreatitis. Intensive Care Med..

[52] Rau B., Steinbach G., Gansauge F., Mayer J.M., Grünert A., Beger H.G. (1997). The potential role of procalcitonin and interleukin 8 in the prediction of infected necrosis in acute pancreatitis. Gut.

[53] Khanna A.K., Meher S., Prakash S., Tiwary S.K., Singh U., Srivastava A., Dixit V.K. (2013). Comparison of Ranson, Glasgow, MOSS, SIRS, BISAP, APACHE-II, CTSI scores, IL-6, CRP, and procalcitonin in predicting severity, organ failure, pancreatic necrosis, and mortality in acute pancreatitis. HPB Surg..

[54] He T., Zhang Y., Wen X., Zhang Z. (2026). Procalcitonin for diagnosing infected pancreatic necrosis: A systematic review and meta-analysis. BMC Gastroenterol..

[55] Tarján D., Szalai E., Lipp M., Verbói M., Kói T., Erőss B., Teutsch B., Faluhelyi N., Hegyi P., Mikó A. (2024). Persistently high procalcitonin and C-reactive protein are good predictors of infection in acute necrotizing pancreatitis: A systematic review and meta-analysis. Int. J. Mol. Sci..

[56] Siriwardena A.K., Jegatheeswaran S., Mason J.M., PROCAP Investigators (2022). A procalcitonin-based algorithm to guide antibiotic use in patients with acute pancreatitis (PROCAP): A single-centre, patient-blinded, randomised controlled trial. Lancet Gastroenterol. Hepatol..

[57] Valentin C., Le Cosquer G., Tuyeras G., Culetto A., Barange K., Hervieu P.E., Carrère N., Muscari F., Mokrane F., Otal P. (2024). Step-up approach for the treatment of infected necrotising pancreatitis: Real-life data from a single-centre experience with long-term follow-up. BMC Gastroenterol..

[58] Lord J.M., Midwinter M.J., Chen Y.F., Belli A., Brohi K., Kovacs E.J., Koenderman L., Kubes P., Lilford R.J. (2014). The systemic immune response to trauma: An overview of pathophysiology and treatment. Lancet.

[59] Guisasola M.C., Alonso B., Bravo B., Vaquero J., Chana F. (2018). An overview of cytokines and heat shock response in polytraumatized patients. Cell Stress Chaperones.

[60] Wojtaszek M., Staśkiewicz G., Torres K., Jakubowski K., Rácz O., Cipora E. (2014). Changes of procalcitonin level in multiple trauma patients. Anaesthesiol. Intensive Ther..

[61] AlRawahi A.N., AlHinai F.A., Doig C.J., Ball C.G., Dixon E., Xiao Z., Kirkpatrick A.W. (2019). The prognostic value of serum procalcitonin measurements in critically injured patients: A systematic review. Crit. Care.

[62] Meisner M., Adina H., Schmidt J. (2006). Correlation of procalcitonin and C-reactive protein to inflammation, complications, and outcome during the intensive care unit course of multiple-trauma patients. Crit. Care.

[63] Moore F.A., Sauaia A., Moore E.E., Haenel J.B., Burch J.M., Lezotte D.C. (1996). Postinjury multiple organ failure: A bimodal phenomenon. J. Trauma..

[64] Maruna P., Nedelníková K., Gürlich R. (2000). Physiology and genetics of procalcitonin. Physiol. Res..

[65] Ramasawmy D., Pillay M., Hardcastle T.C. (2021). Correlation of procalcitonin to positive blood culture results in a sample of South African trauma ICU patients between 2016 and 2017. Eur. J. Trauma Emerg. Surg..

[66] Li M., Qin Y.J., Zhang X.L., Zhang C.H., Ci R.J., Chen W., Hu D.-Z., Dong S.-M. (2024). A biomarker panel of C-reactive protein, procalcitonin and serum amyloid A is a predictor of sepsis in severe trauma patients. Sci. Rep..

[67] Sciuto A., Merola G., De Palma G.D., Sodo M., Pirozzi F., Bracale U.M., Bracale U. (2018). Predictive factors for anastomotic leakage after laparoscopic colorectal surgery. World J. Gastroenterol..

[68] Bezzerri V., Putignani L., Mantuano E., Polini A., Navarini L., Vomero M., Corberi E., Miacci V., Papuc P.E., Schiavone V. (2025). A focus on inflammatory and bacterial biomarkers in secondary peritonitis. Cells.

[69] Gbinigie O.A., Onakpoya I.J., Richards G.C., Spencer E.A., Koshiaris C., Bobrovitz N., Heneghan C.J. (2019). Biomarkers for diagnosing serious bacterial infections in older outpatients: A systematic review. BMC Geriatr..

[70] Bartoletti M., Antonelli M., Bruno Blasi F.A., Casagranda I., Chieregato A., Fumagalli R., Girardis M., Pieralli F., Plebani M., Rossolini G.M. (2018). Procalcitonin-guided antibiotic therapy: An expert consensus. Clin. Chem. Lab. Med..

[71] Giaccaglia V., Salvi P.F., Antonelli M.S., Nigri G., Pirozzi F., Casagranda B., Giacca M., Corcione F., de Manzini N., Balducci G. (2016). Procalcitonin reveals early dehiscence in colorectal surgery: The PREDICS study. Ann. Surg..

[72] Xiong Z., Liu J., Chen L., Yang Y., Zhao S. (2025). Precision leak detection in the golden window: Emerging biomarker strategies for anastomotic integrity monitoring after low anterior resection. Int. J. Surg..

[73] Tan W.J., Ng W.Q., Sultana R., de Souza N.N., Chew M.H., Foo F.J., Tang C.L., Tan W.S. (2018). Systematic review and meta-analysis of the use of serum procalcitonin levels to predict intra-abdominal infections after colorectal surgery. Int. J. Colorectal Dis..

[74] Cousin F., Ortega-Deballon P., Bourredjem A., Doussot A., Giaccaglia V., Fournel I. (2016). Diagnostic accuracy of procalcitonin and C-reactive protein for the early diagnosis of intra-abdominal infection after elective colorectal surgery: A meta-analysis. Ann. Surg..

[75] Giaccaglia V., Salvi P.F., Cunsolo G.V., Sparagna A., Antonelli M.S., Nigri G., Balducci G., Ziparo V. (2014). Procalcitonin, as an early biomarker of colorectal anastomotic leak, facilitates enhanced recovery after surgery. J. Crit. Care.

[76] Zawadzki M., Czarnecki R., Rzaca M., Obuszko Z., Velchuru V.R., Witkiewicz W. (2016). C-reactive protein and procalcitonin predict anastomotic leaks following colorectal cancer resections—A prospective study. Wideochirurgia I Inne Tech. Maloinwazyjne.

[77] Ramírez-Giraldo C., Pesce A., González-Muñoz A., Navarro-Pulido N., Ochoa-Patarroyo M., Vallejo-Soto J.C., Figueroa-Avendaño C., Isaza-Restrepo A. (2025). Diagnostic performance of procalcitonin for detecting anastomotic leak in older adults with colorectal cancer: A delayed type cross-sectional study. Surgery.

[78] Villegas-Coronado L., Villegas-Coronado K., Urrea-Quezada A., Villegas-Coronado D. (2024). Procalcitonin as an early marker of colorectal anastomotic leakage in postoperative colorectal cancer patients: A systematic review and meta-analysis. Cureus.

[79] Jerome E., McPhail M.J., Menon K. (2022). Diagnostic accuracy of procalcitonin and interleukin-6 for postoperative infection in major gastrointestinal surgery: A systematic review and meta-analysis. Ann. R Coll. Surg. Engl..

[80] Chambliss A.B., Patel K., Colón-Franco J.M., Hayden J., Katz S.E., Minejima E., Woodworth A. (2023). AACC Guidance Document on the Clinical Use of Procalcitonin. J. Appl. Lab. Med..

[81] Kretzschmar M., Krüger A., Schirrmeister W. (2001). Procalcitonin following elective partial liver resection—Origin from the liver?*. Acta Anaesthesiol. Scand..

[82] Foulon N., Haeger S.M., Okamura K., He Z., Park B.D., Budnick I.M., Madison D., Kennis M., Blaine R., Miyazaki M. (2025). Procalcitonin levels in septic and nonseptic subjects with AKI and ESKD prior to and during continuous kidney replacement therapy (CKRT). Crit. Care.

[83] Lippi G., Salvagno G.L., Gelati M., Pucci M., Lo Cascio C., Demonte D., Faggian D., Plebani M. (2019). Two-center comparison of 10 fully-automated commercial procalcitonin (PCT) immunoassays. Clin. Chem. Lab. Med..

