# Supplementary material for: Procalcitonin-Informed Decision-Making in Abdominal and Emergency Surgery: A WSES Position Paper
Source: Antibiotics (Basel). 2026 Oct 21;15(10):1042. doi: 10.3390/antibiotics15101042 (PMC13700824; doi:10.3390/antibiotics15101042)
Supplement: Supplementary file 1 [file antibiotics-15-01042-s001.zip › antibiotics-4605790-supplementary.pdf]

Table S1: Recommendation 1 Diagnosis and reassessment of intra-abdominal infection and selected evidence

Reference and study	Design and population	PCT assessment	Comparator or reference standard; outcome	Main findings and clinical implications
[16] Simon 2004<br>Corrected in 2005	Systematic review/meta-analysis<br>12 studies; 1,386 patients; mixed infection settings	Assay: Varied across included studies<br>Timing: At clinical suspicion<br>Threshold: Varied across included studies	Comparator: CRP<br>Outcome: Diagnostic accuracy for bacterial infection	Findings: Corrected pooled estimates for bacterial infection versus noninfectious inflammation: PCT sensitivity 85% (95% CI 76–91), specificity 83% (68–92); CRP 78% (70–85) and 60% (38–79), respectively.<br>Clinical implication: Background comparison; not an IAI-specific management study.
[17] Ozen 2025	Single-centre retrospective study<br>245 acute-abdomen admissions; 167 in the four abdominal disease groups	Timing: Samples within 2 h of admission; PCT assayed after recruitment, with clinicians blinded<br>Threshold: Laboratory reference ≥0.04 μg/L; ≥0.5 μg/L also examined	Comparator: Conventional inflammatory markers<br>Outcome: IAI severity grading	Findings: Higher PCT in selected severe disease subgroups, including complicated diverticulitis and severe cholecystitis.<br>Clinical implication: Supports contextual severity assessment; does not validate a universal diagnostic threshold.
[18] Covino 2021	Retrospective propensity-matched cohort<br>3,429 emergency-department patients with IAI	Timing: Early emergency-department measurement	Comparator: No early PCT assessment; propensity-matched groups<br>Outcome: Major clinical outcomes	Findings: Early PCT measurement was not associated with improved major clinical outcomes after propensity matching.<br>Clinical implication: Early PCT measurement was not associated with improved major clinical outcomes in this cohort.
[19] Yu 2013	Systematic review/meta-analysis<br>7 studies; 1,011 suspected cases, 636 confirmed appendicitis cases	Assay: Varied across included studies<br>Timing: At diagnostic evaluation<br>Threshold: Varied across included studies	Comparator: CRP and white-cell count<br>Outcome: Appendicitis and complicated appendicitis	Findings: For complicated appendicitis, PCT sensitivity 62% (95% CI 33–84) and specificity 94% (90–96).<br>Clinical implication: High specificity may support suspicion; sensitivity is insufficient for exclusion.
[20] Dale 2022	Systematic review<br>16 primary appendicitis studies	Assay: Varied across included studies<br>Timing: At diagnostic evaluation<br>Threshold: Varied across included studies	Comparator: Clinical assessment and inflammatory markers<br>Outcome: Acute appendicitis diagnosis	Findings: Findings varied with appendicitis severity; PCT had a limited standalone role in acute appendicitis diagnosis.<br>Clinical implication: Supports interpretation alongside clinical assessment and other investigations.
[21] Jeger 2017	Prospective diagnostic cohort<br>115 patients with acute diverticulitis	Timing: Admission and day 2; highest value used<br>Threshold: 0.1 μg/L (=0.1 ng/mL) using highest of days 1–2	Comparator: CT classification<br>Outcome: Uncomplicated versus complicated diverticulitis	Findings: Sensitivity 81% and specificity 91% for highest value across days 1–2.<br>Clinical implication: PCT may assist differentiation of complicated from uncomplicated diverticulitis; imaging remains necessary when complications are suspected.

Reference and study	Design and population	PCT assessment	Comparator or reference standard; outcome	Main findings and clinical implications
[22] Schena 2024	Retrospective cohort<br>503 adults with acute left-sided colonic diverticulitis	Timing: At admission	Comparator: Clinical and imaging severity assessment<br>Outcome: Severity, prognosis and need for surgery	Findings: Elevated admission PCT was independently associated with complicated disease, surgery, sigmoidectomy without anastomosis and mortality.<br>Clinical implication: Observational risk stratification; no demonstrated benefit from PCT-triggered management.
[23] Yaow 2023	Systematic review; 5 studies<br>Acute cholecystitis; 588 patients	Assay: Varied across included studies<br>Timing: At initial assessment<br>Threshold: Varied across included studies	Comparator: Clinical severity grading<br>Outcome: Severity of acute cholecystitis	Findings: Evidence for discrimination between acute cholecystitis severity grades was inconsistent.<br>Clinical implication: Potential adjunct to severity assessment; cannot replace established clinical and imaging criteria.
[24] Arbutina 2022	Prospective cohort<br>100 adults aged 18–70 with diffuse secondary peritonitis	Timing: Preoperative and postoperative assessments	Comparator: Other inflammatory biomarkers<br>Outcome: Early abdominal-sepsis diagnosis	Findings: CRP and PCT helped distinguish sepsis from SIRS preoperatively; postoperative diagnostic accuracy was limited, with SAA performing better on day 1.<br>Clinical implication: Utility depends on sampling time; no reliable standalone postoperative diagnostic rule.
[25] Yang 2014	Systematic review/meta-analysis<br>18 studies; 1,827 patients; spontaneous or peritoneal dialysis-related peritonitis	Assay: Varied across included studies<br>Timing: At suspected peritonitis<br>Threshold: Varied across included studies	Comparator: Reference diagnosis/culture<br>Outcome: Diagnostic accuracy for bacterial peritonitis	Findings: Pooled sensitivity 83%, specificity 92%, AUC 0.94.<br>Clinical implication: Applicability to secondary surgical peritonitis is limited.
[26] Perrella 2022	Observational postoperative study<br>Infections after major non-emergency abdominal surgery; 260 patients	Timing: Serial postoperative assessment	Comparator: CRP and clinical course<br>Outcome: Antibiotic response and outcome	Findings: CRP, but not PCT, predicted response/outcome in the reported analysis.<br>Clinical implication: This study did not demonstrate an advantage of PCT over CRP.
[30] Wacker 2013	Systematic review/meta-analysis<br>30 studies; 3,244 adults with suspected sepsis	Assay: Varied across included studies<br>Timing: At sepsis assessment<br>Threshold: Varied across included studies	Comparator: Clinical reference standard<br>Outcome: Sepsis diagnostic accuracy	Findings: Pooled sensitivity 77%, specificity 79%, AUC 0.85.<br>Clinical implication: These estimates concern sepsis diagnosis in mixed populations and do not validate IAI-specific management decisions.

Table S2: Recommendation 2 Antibiotic discontinuation after adequate source control; selected evidence

Reference and study	Design and population	PCT assessment	Comparator or reference standard; outcome	Main findings and clinical implications
[28] Sawyer 2015 (STOP-IT)	Randomized controlled trial<br>518 patients with complicated IAI after adequate source control	Assay: No PCT intervention<br>Timing: Fixed course versus physiological-resolution strategy<br>Threshold: Not applicable	Comparator: About 4 versus 8 days of antibiotics<br>Outcome: Composite surgical-site infection, recurrent IAI or death	Findings: The composite outcome occurred in 21.8% of patients receiving approximately 4 days of antibiotics and 22.3% receiving approximately 8 days.<br>Clinical implication: Shows short treatment can be sufficient after source control; cannot establish PCT benefit.
[29] de Jong 2016 (SAPS)	Randomized open-label trial<br>1,546 critically ill patients analyzed	Timing: Daily during antibiotic treatment<br>Threshold: Consider discontinuation if PCT <0.5 ng/mL or ≥80% decrease from peak	Comparator: Usual-care antibiotic decisions<br>Outcome: Antibiotic duration; 28-day mortality	Findings: Median antibiotic duration was 5 days with PCT guidance versus 7 days with usual care; 28-day mortality was 20% versus 25%, respectively.<br>Clinical implication: Supports PCT-informed antibiotic discontinuation in critically ill patients; application to surgical IAI requires clinical assessment of stability and source control.
[31] Wirz 2018	Individual-patient-data meta-analysis<br>11 ICU randomized trials; 4,482 patients	Assay: Varied across included studies<br>Timing: Serial measurements during treatment<br>Threshold: Varied across included studies	Comparator: Usual care<br>Outcome: Antibiotic duration; clinical outcomes	Findings: Antibiotic duration was 9.3 days with PCT guidance versus 10.4 days with usual care; adjusted difference −1.19 days.<br>Clinical implication: Supports shorter antibiotic courses in mixed ICU populations; applicability to surgical IAI requires clinical judgment.
[32] Pepper 2019	Systematic review/meta-analysis<br>16 randomized trials; 5,158 randomized, 5,000 analyzed	Assay: Varied across included studies<br>Timing: Serial treatment monitoring<br>Threshold: Varied across included studies	Comparator: Usual care<br>Outcome: Antibiotic duration and mortality	Findings: Antibiotic duration MD −1.31 days (95% CI −2.27 to −0.35); mortality RR 0.89 (0.83–0.97). Authors rated evidence as low certainty; mortality benefit was not significant in the sepsis subset.<br>Clinical implication: Supports discontinuation algorithms; survival benefit remains uncertain.
[33] Papp 2023	Systematic review/meta-analysis<br>26 randomized trials; 9,048 critically ill patients	Assay: Varied across included studies<br>Timing: Serial treatment monitoring<br>Threshold: Varied across included studies	Comparator: Usual care<br>Outcome: Antibiotic duration and survival	Findings: Antibiotic duration MD −1.79 days (95% CI −2.65 to −0.92); 28-day mortality OR 0.84 (0.74–0.95); recurrent infection OR 1.36 (1.10–1.68).<br>Clinical implication: PCT guidance reduced antibiotic exposure but was associated with more recurrent infections. Included trials may overlap with other meta-analyses listed here.
[34] Huang 2014	Prospective cohort with propensity-matched historical controls<br>30 PCT patients and 60 controls after emergency	Assay: BRAHMS PCT KRYPTOR<br>Timing: Preoperatively and POD1, 3, 5, 7; thereafter as needed<br>Threshold: <1 ng/mL or ≥80% decline from POD1, with resolution of clinical signs	Comparator: Historical usual-care treatment<br>Outcome: Antibiotic duration	Findings: Median antibiotic duration was 3.4 days with PCT guidance versus 6.1 days in historical controls (p<0.001).<br>Clinical implication: Supports the feasibility of PCT-informed antibiotic discontinuation after surgery for secondary peritonitis; the nonrandomized design limits causal inference.

Reference and study	Design and population	PCT assessment	Comparator or reference standard; outcome	Main findings and clinical implications
	surgery for secondary peritonitis			
[35] Maseda 2015	Multicentre retrospective study<br>121 surgical-ICU patients with controlled secondary peritonitis	Timing: Serial after source control	Comparator: Conventional antibiotic decisions<br>Outcome: Antibiotic duration; mortality/length of stay	Findings: Mean antibiotic duration was 5.1 ± 2.1 days with PCT guidance versus 10.2 ± 3.7 days without PCT guidance ($p<0.001$). Mortality and length of stay did not differ significantly.<br>Clinical implication: Potential stewardship benefit; retrospective design limits causal inference.
[36] Slieker 2017	Randomized controlled trial<br>162 patients after surgery for peritonitis	Timing: Surgery/POD1, POD3, then every 48 h<br>Threshold: Continue through POD3; stop if <1 ng/mL at POD3; subsequently <0.25 ng/mL or ≥80% fall from baseline, with clinical assessment	Comparator: Standard antibiotic decisions<br>Outcome: Antibiotic duration	Findings: Median antibiotic duration with PCT guidance versus standard care was 8 versus 10 days overall ($p=0.714$), and 7 versus 10 days in the gastrointestinal-perforation subgroup ($p=0.065$).<br>Clinical implication: PCT guidance did not significantly reduce overall antibiotic duration in this surgical peritonitis trial.
[42] Sartelli 2021	Expert consensus/review<br>Surgical pathway; no primary patient sample	Assay: Not applicable<br>Timing: Across surgical pathway<br>Threshold: Narrative use; no validated universal cutoff	Comparator: Prior literature and expert consensus<br>Outcome: Antibiotic use across surgery	Findings: No primary effect estimate.<br>Clinical implication: Provides expert interpretation of antibiotic stewardship across the surgical pathway, rather than an independent patient dataset.

Table S3: Recommendation 3 Reassessment for suspected source-control failure; selected evidence

Reference and study	Design and population	PCT assessment	Comparator or reference standard; outcome	Main findings and clinical implications
[39] van Ruler 2007 (RELAP)	Randomized controlled trial<br>232 patients with severe secondary peritonitis	Assay: No PCT intervention<br>Timing: Not applicable<br>Threshold: Not applicable	Comparator: On-demand versus planned relaparotomy<br>Outcome: Death or major peritonitis-related morbidity; resource use	Findings: The composite outcome occurred in 57% with on-demand versus 65% with planned relaparotomy. First relaparotomies were negative in 31% versus 66%, respectively.<br>Clinical implication: Supports an on-demand relaparotomy strategy but does not evaluate PCT-based criteria for reintervention.
[41] Assfalg 2016	Prospective focus-index validation<br>234 patients with secondary peritonitis	Timing: First sample 12–24 h after operation; ratio postoperative day 1/day 2<br>Threshold: Focus index = PCT(POD1) / PCT(POD2); >1.1 for successful focus elimination	Comparator: Clinical focus-control assessment<br>Outcome: Prediction of successful elimination	Findings: After the data-cleaning procedure reported by the authors, a focus index >1.1 identified successful source control with 93% sensitivity and 71% specificity.<br>Clinical implication: An early decline in PCT may support assessment of source-control success when interpreted alongside clinical findings.
[43] Novotny 2009	Observational surgical cohort<br>104 patients with abdominal sepsis/secondary peritonitis	Timing: Postoperative day 1 and day 2<br>Threshold: PCT ratio = PCT(POD1) / PCT(POD2); <1.03 raises concern for failure	Comparator: Observed treatment success/failure<br>Outcome: Detection of unsuccessful surgical treatment	Findings: For unsuccessful treatment, sensitivity 95%, specificity 63% using the 1.03 ratio cut-off.<br>Clinical implication: Early PCT kinetics may inform reassessment, but specificity is insufficient to guide reintervention alone.
[44] Jung 2013	Prospective observational cohort<br>101 perioperative abdominal-infection patients with septic shock	Timing: Day 0 to day 5<br>Threshold: Target <0.5 ng/mL or ≥80% reduction evaluated	Comparator: Observed clinical treatment response<br>Outcome: Prediction of treatment response	Findings: A decline of ≥80% from peak PCT predicted treatment success with 63% sensitivity, 43% specificity and 56% accuracy.<br>Clinical implication: Serial PCT alone did not reliably predict treatment response in patients with abdominal infection and septic shock.

Table S4: Recommendation 4 Acute pancreatitis and infected pancreatic necrosis; selected evidence

Reference and study	Design and population	PCT assessment	Comparator or reference standard; outcome	Main findings and clinical implications
[49] Mofidi 2009	Systematic review/meta-analysis<br>Infected necrosis: 7 studies, 264 patients	Assay: Varied across included studies<br>Timing: At variable times in acute pancreatitis<br>Threshold: Varied across included studies	Comparator: Reference diagnosis of infected necrosis<br>Outcome: Diagnostic accuracy for infected pancreatic necrosis	Findings: Pooled sensitivity 0.80, specificity 0.91, AUC 0.91.<br>Clinical implication: Supports suspicion of IPN; threshold and timing vary among studies.
[51] Rau 2000	Clinical observational study<br>61 patients: 22 edematous pancreatitis, 18 sterile necrosis, 21 infected necrosis	Assay: Immunoluminometry<br>Timing: Serial testing over 14 days<br>Threshold: ≥1.8 ng/mL on at least two consecutive days	Comparator: Clinical/imaging and infection diagnosis<br>Outcome: Prediction of infected necrosis	Findings: Sensitivity 95%, specificity 88%, accuracy 90% for infected necrosis.<br>Clinical implication: Repeated elevation supports suspicion; performance is not that of a single admission measurement.
[52] Rau 1997	Prospective biomarker cohort<br>50 patients: 18 edematous, 14 sterile necrosis, 18 infected necrosis	Assay: Chemoluminescent immunoassay<br>Timing: Daily for two weeks after admission<br>Threshold: 1.8 ng/mL reached on at least two days	Comparator: IL-8; clinical infection status<br>Outcome: Prediction of infected necrosis	Findings: Sensitivity 94%, specificity 91%.<br>Clinical implication: Supports interpretation alongside clinical and imaging findings; the threshold requires validation in other populations.
[53] Khanna 2013	Comparative biomarker/score study<br>72 acute pancreatitis patients; 31 severe cases, 17 with necrosis, 9 deaths	Timing: During initial severity assessment	Comparator: Ranson, Glasgow, MOSS, SIRS, BISAP, APACHE-II, CTSI, IL-6, CRP<br>Outcome: Severity, organ failure, necrosis, mortality	Findings: Compared PCT with clinical scores and other biomarkers for severity and adverse outcomes.<br>Clinical implication: Prognostic comparisons do not establish PCT-guided management utility.
[54] He 2026	Systematic review/meta-analysis<br>16 PCT diagnostic studies; 2,156 patients, 512 IPN	Assay: Varied across included studies<br>Timing: Admission and serial assessments<br>Threshold: Study-specific; serial versus initial value analyzed	Comparator: Reference diagnosis of IPN<br>Outcome: IPN diagnostic accuracy	Findings: Sensitivity 0.82 (95% CI 0.75–0.87), specificity 0.84 (0.78–0.89), AUC 0.90 (0.87–0.92); serial AUC 0.93 vs admission 0.85.<br>Clinical implication: Serial monitoring showed higher reported discrimination; improved management or clinical outcomes were not demonstrated.
[55] Tarján 2024	Systematic review/meta-analysis<br>8 studies in meta-analysis; 758 patients, 324 with IPN	Assay: Varied across included studies<br>Timing: Within versus after 72 hours<br>Threshold: Persistent high PCT; thresholds varied	Comparator: CRP and infection reference diagnosis<br>Outcome: Infection in acute necrotizing pancreatitis	Findings: PCT AUC 0.69 (95% CI 0.60–0.78) within 72 h and 0.86 (reported CI 0.60–1.11) thereafter. The latter estimate is imprecise, with a reported upper bound above 1.<br>Clinical implication: Later measurements may discriminate better; early normal PCT cannot exclude subsequent infection.

Reference and study	Design and population	PCT assessment	Comparator or reference standard; outcome	Main findings and clinical implications
[56] Siriwardena 2022 (PROCAP)	Patient-blinded randomized controlled trial<br>260 patients with acute pancreatitis (132 PCT, 128 usual care)	Timing: Days 0, 4, 7, then weekly; before empirical antibiotic decisions<br>Threshold: <1 ng/mL, stop/do not start; ≥1 ng/mL, start/continue, within the trial algorithm	Comparator: Usual-care antibiotic decisions<br>Outcome: Antibiotic prescribing	Findings: Antibiotics were prescribed to 59 patients in the PCT group versus 79 with usual care (45% versus 63%, as published); adjusted risk difference −15.6 percentage points (95% CI −27.0 to −4.2).<br>Clinical implication: Supports protocol-based antibiotic stewardship in acute pancreatitis; it does not establish diagnostic accuracy for IPN or justify delaying indicated source control.
[57] Valentin 2024	Single-centre retrospective observational series<br>Patients with infected necrotizing pancreatitis	Timing: During step-up treatment<br>No PCT-directed intervention	Comparator: Clinical/imaging response<br>Outcome: Step-up treatment outcomes	Findings: Evaluated feasibility, infection resolution, procedure morbidity, mortality and long-term outcomes of step-up treatment.<br>Clinical implication: Provides context for source-control management of IPN rather than evidence of PCT diagnostic accuracy or clinical utility.

Table S5: Recommendation 5 Infectious complications after trauma; selected evidence

Reference and study	Design and population	PCT assessment	Comparator or reference standard; outcome	Main findings and clinical implications
[60] Wojtaszek 2014	Prospective observational study<br>45 trauma patients, including an isolated CNS-injury group	Assay: Enzyme-linked immunoassay<br>Timing: Serial after injury, including 24 h	Comparator: Injury pattern, time from injury and survival<br>Outcome: Post-traumatic PCT trajectory	Findings: PCT increased at 24 h and subsequently declined; levels varied with injury pattern and were higher in nonsurvivors.<br>Clinical implication: Early post-traumatic elevation is not specific for infection.
[61] AlRawahi 2019	Systematic review<br>19 observational studies; 4,146 critically injured adults	Assay: Varied across included studies<br>Timing: First 1–2 days and subsequent serial measurements<br>Threshold: Varied across included studies	Comparator: Observed infection/sepsis and other biomarkers<br>Outcome: Sepsis and complications	Findings: Initial PCT higher in patients with sepsis in 12 of 16 reporting studies; no pooled effect due to heterogeneity.<br>Clinical implication: Associations with sepsis support further evaluation, but do not establish a validated diagnostic pathway based on PCT alone.
[62] Meisner 2006	Prospective ICU cohort<br>90 multiple-trauma patients	Timing: Serial intensive-care course<br>Threshold: Early PCT >1 ng/mL evaluated	Comparator: CRP and clinical complications<br>Outcome: Inflammation, complications, outcome	Findings: Early PCT >1 ng/mL was associated with infection, sepsis, prolonged ICU treatment and poor outcome.<br>Clinical implication: Supports contextual assessment of kinetics, not a universal infection threshold.
[65] Ramasawmy 2021	Retrospective trauma-ICU study<br>149 trauma-ICU patients	Timing: Around blood-culture assessment<br>Threshold: >2 and >31 ng/mL explored for differing sensitivity/specificity	Comparator: Blood-culture positivity<br>Outcome: Positive blood cultures	Findings: Median PCT was 30.5 ng/mL in patients with positive blood cultures versus 6.6 ng/mL in those with negative cultures (p=0.002).<br>Clinical implication: An association with positive blood cultures does not establish improved outcomes from PCT-informed treatment decisions.
[66] Li 2024	Prospective biomarker-panel study<br>100 severe-trauma patients; 52 developed sepsis	Assay: Commercial ELISA kits<br>Timing: Daily sampling; five selected time points around sepsis onset or after admission in nonseptic patients<br>Threshold: Model-specific bioscore	Comparator: CRP, serum amyloid A, combined panel<br>Outcome: Sepsis prediction	Findings: PCT AUC 0.77 (95% CI 0.68–0.86), CRP 0.82 (0.73–0.91), combined PCT+CRP+SAA 0.89 (0.82–0.95).<br>Clinical implication: The combined panel showed promising discrimination. PCT alone did not outperform CRP; external validation and clinical benefit remain unestablished.

Table S6: Recommendation 6 Postoperative anastomotic leak; selected evidence.

Reference and study	Design and population	PCT assessment	Comparator or reference standard; outcome	Main findings and clinical implications
[72] Giaccaglia 2016 (PREDICS)	Prospective multicentre cohort<br>504 elective colorectal-cancer patients; 28 leaks	Timing: Postoperative days 3 and 5<br>Threshold: <2.7 ng/mL POD3; <2.3 ng/mL POD5	Comparator: CRP and clinical evaluation<br>Outcome: Anastomotic leak exclusion	Findings: NPV 96.9% POD3 and 98.3% POD5; PCT+CRP POD5 AUC 0.901.<br>Clinical implication: Low PCT may support discharge assessment in clinically well patients; the high NPV should be interpreted in the context of the low leak prevalence.
[73] Xiong 2025	Narrative biomarker review<br>Low anterior resection; no primary patient sample	Assay: Not applicable<br>Timing: Postoperative monitoring window<br>Threshold: Narrative thresholds	Comparator: Other biomarkers and standard assessment<br>Outcome: Anastomotic-integrity monitoring	Findings: No independent primary effect estimate.<br>Clinical implication: Provides an overview of biomarkers for anastomotic leakage rather than an independent patient dataset.
[74] Tan 2018	Systematic review/meta-analysis<br>8 studies; 1,629 colorectal surgical patients	Assay: Varied across included studies<br>Timing: Postoperative days 3–5<br>Threshold: Reported optimum 1.45, 1.28, 1.26 ng/mL on POD3, 4, 5	Comparator: Clinical diagnosis of postoperative IAI<br>Outcome: Postoperative IAI including leak	Findings: AUC POD3 0.83 (95% CI 0.78–0.88), POD4 0.79 (0.64–0.93), POD5 0.94 (0.91–0.97); POD5 sensitivity 0.78 (0.65–0.89), specificity 0.88 (0.85–0.90).<br>Clinical implication: These estimates concern postoperative IAI and are not specific to anastomotic leakage.
[75] Cousin 2016	Systematic review/meta-analysis<br>11 studies; 2,692 elective colorectal patients	Assay: Varied across included studies<br>Timing: Postoperative days 2–5<br>Threshold: Varied across included studies	Comparator: CRP<br>Outcome: Diagnostic accuracy for postoperative IAI	Findings: Comparative synthesis found no consistent diagnostic advantage of PCT over CRP.<br>Clinical implication: Incremental value beyond CRP remains uncertain.
[76] Giaccaglia 2014	Prospective colorectal cohort<br>99 patients; 7 anastomotic leaks	Timing: Postoperative days 1, 3 and 5	Comparator: CRP, white blood cells<br>Outcome: Anastomotic leak	Findings: PCT NPV 96.7% on POD3 and POD5; PCT+CRP AUC 0.87 POD3 and 0.94 POD5.<br>Clinical implication: Combining PCT and CRP showed promising discrimination, but only seven leaks occurred.
[77] Zawadzki 2016	Prospective colorectal-cancer cohort<br>55 elective resections; 5 anastomotic leaks	Timing: Preoperatively, 8 h after incision, POD1 and POD3<br>Threshold: PCT 3.83 ng/mL on POD3	Comparator: CRP and clinical diagnosis<br>Outcome: Anastomotic leak prediction	Findings: At POD3, PCT sensitivity 75%, specificity 100%; CRP at 245.64 mg/L had sensitivity 100%, specificity 98%.<br>Clinical implication: Small cohort with only five leaks; thresholds require validation.
[78] Ramírez-Giraldo 2025	Delayed cross-sectional diagnostic study<br>338 older adults with colorectal cancer	Timing: Single sample on POD3<br>Threshold: 0.61 ng/mL by Youden index	Comparator: Reference anastomotic-leak diagnosis<br>Outcome: Overall and severe (grade C) leak detection	Findings: Overall AUC 0.68 (95% CI 0.58–0.78); sensitivity 69%, specificity 62% at 0.61 ng/mL. AUC 0.81 for grade C leaks.<br>Clinical implication: Limited overall discrimination; a single result cannot reliably exclude leak.

Reference and study	Design and population	PCT assessment	Comparator or reference standard; outcome	Main findings and clinical implications
[79] Villegas-Coronado 2024	Systematic review/meta-analysis<br>17 articles, 3,813 participants (277 leaks); cancer-only meta-analysis used 3 studies POD2 and 5 POD3	Assay: 4 CLIA, 4 ELISA, 2 ELFA; 7 unspecified<br>Timing: Postoperative days 2 and 3<br>Threshold: Varied across included studies	Comparator: Leak versus no leak<br>Outcome: Group-level PCT differences	Findings: Cancer-only pooled mean difference 1.188 ng/mL POD2 (95% CI 0.002–2.373) and 2.048 POD3 (0.161–3.935); statistical significance unstable on leave-one-out analysis.<br>Clinical implication: Differences between groups do not establish a validated diagnostic threshold; the pooled results were sensitive to exclusion of individual studies.
[80] Jerome 2022	Systematic review/meta-analysis<br>Major gastrointestinal surgery; PCT analysis: 10 studies, 1,611 patients	Assay: Varied across included studies<br>Timing: Variable postoperative sampling<br>Threshold: Varied across included studies	Comparator: IL-6 and clinical infection diagnosis<br>Outcome: Postoperative infection/sepsis	Findings: Pooled PCT sensitivity 72% (95% CI 66–78), specificity 62% (59–64), summary ROC AUC 0.766; heterogeneous timing and cutoffs.<br>Clinical implication: Moderate diagnostic performance; not leak-specific evidence.

Table notes

Correction to reference [16]: Simon et al. (2004; 39:206–17). Clinical Infectious Diseases. 2005;40(9):1386–1388. doi:10.1086/429520. The corrected estimates are used in Table S1.

These tables summarize selected studies relevant to the six recommendations. Clinical implications describe the relevance and limitations of each study and are not a formal assessment of evidence certainty. Confidence intervals are 95% where specified. Study thresholds are reported for interpretation of the cited evidence and are not universal treatment criteria. Evidence from elective surgery or mixed ICU populations may not be directly applicable to emergency surgical patients.

Abbreviations

PCT, procalcitonin; CRP, C-reactive protein; IAI, intra-abdominal infection; IPN, infected pancreatic necrosis; POD, postoperative day; ICU, intensive care unit; ED, emergency department; CNS, central nervous system; CT, computed tomography; SIRS, systemic inflammatory response syndrome; SAA, serum amyloid A; IL, interleukin; AUC, area under the receiver operating characteristic curve; ROC, receiver operating characteristic; NPV, negative predictive value; CI, confidence interval; MD, mean difference; OR, odds ratio; RR, risk ratio; WSES, World Society of Emergency Surgery.

Assays: CLIA, chemiluminescence immunoassay; ELISA, enzyme-linked immunosorbent assay; ELFA, enzyme-linked fluorescent assay. Severity scores: APACHE II, Acute Physiology and Chronic Health Evaluation II; BISAP, Bedside Index for Severity in Acute Pancreatitis; CTSI, computed tomography severity index; MOSS, Multiple Organ System Score. Ranson and Glasgow refer to pancreatitis severity scores.
